# Enhanced Plant Growth on Simulated Martian Regolith via Water Chemistry Optimisation: The Role of RONS and Nano/Micro-Bubbles

**DOI:** 10.3390/ijms26178318

**Published:** 2025-08-27

**Authors:** Syamlal Sasi, Priyanka Prakash, Steve Hayden, David Dooley, Richard Poiré, Tao Hu, Janith Weerasinghe, Igor Levchenko, Karthika Prasad, Katia Alexander

**Affiliations:** 1 School of Engineering, ANU College of Systems and Society, The Australian National University, Canberra, ACT 2600, Australia; syamlal.sasi@anu.edu.au (S.S.); priyanka.priyanka1@anu.edu.au (P.P.); janith.adikarammudiyanselage@anu.edu.au (J.W.); 2 Aquapulse-Product Development, Clear World Water Technology Limited, Stanmore, Middlesex HA7 4PX, UK; steve.hayden@aquapulse.tech (S.H.); david.dooley@aquapulse.tech (D.D.); 3 Australian Plant Phenomics Network, The Australian National University, Canberra, ACT 2600, Australia; richard.poire@anu.edu.au (R.P.); tao.hu@anu.edu.au (T.H.); 4 Dipartimento di Fisica, Università di Milano-Bicocca, Piazza della Scienza 3, 20126 Milano, Italy; i.levchenko@post.com

**Keywords:** simulated Martian regolith, nano-/micro-bubbles, cold atmospheric plasma

## Abstract

Development of sustainable agriculture on Mars is a critical step towards its colonisation. However, Martian regolith is coarse-grained, and its mineral profile differs significantly from that of terrestrial arable soil, resulting in poor seed germination success and stunted plant development. This study investigates whether germination success and plant growth can be improved by exposing seeds and plants to water enriched with either i) biochemically active reactive oxygen and nitrogen species generated by atmospheric pressure plasma (PAW) or (ii) nano-/micro-bubbles and minerals such as potassium and calcium extracted from Aquapulse^®^ feldspar (APW), a type of rock that is readily available on Mars, at different stages of the crop lifecycle. As a crop model, microgreen crops of *B. oleracea* and *M. sativa* are chosen for their short growth cycle, low resource requirements, and high nutritional value. For *B. oleracea* crops, soaking of seeds in PAW followed by irrigation with APW led to an increase in germination by ~566.7%, in biomass by 412.4%, and in chlorophyll content by 17.7% compared to crops grown using normal water for seed soaking and irrigation. For *M. sativa* crops, the use of APW for soaking and irrigation yielded an increase of 41.7% in seed germination and 45.2% in crop biomass, whereas the use of PAW for both soaking and irrigation resulted in the greatest improvement in seed germination, 41.7%, when compared to control. These results suggest that, with further optimisation, a regiment of treatment with PAW and APW in place of normal water can be used to address stage-specific challenges of the crop lifecycle in Martian regolith. As amending Martian regolith with a minimum of 1% organic matter is required to promote healthy plant development, further studies should investigate the use of plasma-mediated reforming of biowaste for in situ production of e.g., biochar.

## 1. Introduction

Human colonisation of Mars has gained strong interest, with government agencies such as NASA and private companies such as SpaceX currently working on the development of supply and operational infrastructure, with a longer-term goal of establishing a self-sustaining colony by 2050 [1,2]. In order to realise this ambitious vision, the focus of space engineering has broadened from the technologies necessary for interplanetary travel [3] to include the development of systems capable of recycling and in situ utilisation of local resources such as Martian regolith, water ice, and the atmosphere to reduce the dependence on Earth-derived resources [4,5]. One of the areas that is receiving interest is focused on the use of Martian regolith as a substrate for plant growth [1,6]. Martian regolith consists mostly of weathered basaltic rock, including plagioclase feldspars, pyroxenes, and olivine, and as such may act as a rich source of minerals to sustain plant life. However, as a growth substrate, Martian regolith lacks organic matter and essential nutrients such as nitrogen and phosphorus [7] required for plant growth [8], while at the same time containing increased levels of germination- and growth-retarding compounds, and its water holding capacity is only around 30% of that of terrestrial soils [9]. The combination of unsuitable nutrient profile and poor water transport in unconditioned Martian regolith leads to difficulties in seed germination, root system development, and plant growth [7].

To overcome these challenges, there are several strategies currently being considered. One such strategy is focused on selecting stress-tolerant crops, as they are more likely to thrive under such resource-constrained conditions. For example, salad crops such as microgreens offer a favourable combination of high nutrient density, high harvest index, and lower space and water requirements [10,11], with legume microgreens also considered for their nitrogen fixation potential [12,13]. The stress tolerance and productivity of these crops can be further increased by e.g., subjecting the seeds and plants at different stages of their lifecycle to cold atmospheric plasma, a weakly ionised gas rich in reactive oxygen and nitrogen species (RONS) which can be transferred to and retained in plasma-activated water (PAW). When applied to seeds, both direct plasma and PAW treatments have been shown to increase seed germination [14], including in legumes [15], and promote nutrient uptake and early plant development. These changes may arise as a result of the interactions between plasma-generated RONS and the surface of the seed coat, leading to surface modifications that increase hydrophilicity and facilitate enhanced oxygen permeation and water uptake. In addition, RONS formed during plasma treatment of water may function as signalling molecules, causing early metabolic processes and initiating stress-related signalling pathways for germination [16]. ROS are known to modulate the cell redox state, thereby activating transcription factors such as those in the WRKY, MYB, and NAC families, which take part in plant stress response, seed germination, and root development. These transcription factors play crucial roles in the activation of plant responses to stresses and developmental events [17].

When used for irrigation, PAW can increase the expression of genes related to plant defence and resistance to both biotic (pathogens) and abiotic stress, promote growth and biomass accumulation, and positively interact with soil to optimise its pH and enhance nutrient availability [18,19,20,21]. The relationship between microorganisms and plasma-generated effects are complex. On one end of the spectrum, treatment with direct plasma or PAW can be tailored to effectively decontaminate surfaces and liquids [22,23], including food, enabling their safe consumption and re-use, which is particularly important for closed-loop systems and in cases where resources are constrained. On the other end of the spectrum, plasma treatment has been used to stimulate stress tolerance, biomass accumulation, and metabolic activity in industrial microorganisms [24], which can be exploited in e.g., engineering of soil microbial systems.

With respect to optimising the mineral profile of Martian regolith, another potential strategy may involve the use of other local minerals, such as feldspar, as a source essential minerals like potassium, sodium, and calcium [25,26]. On Earth, feldspars have been used extensively in agriculture as an alternative source of macronutrients such as K and micronutrients such as Ca and Mg, to reduce reliance on chemical fertilisers, thereby lowering the environmental impact and cost of terrestrial agriculture; however, accelerating the release of desired nutrients while simultaneously managing the release of potentially toxic elements such as Al remains a challenge. Proprietary combinations of feldspars, e.g., Aquapulse^®^ [27], have been shown to improve the agricultural productivity of marigolds (+21% in bud and flower development, +29% fresh weight, increased root development), tomatoes (+56% in dry weight), capsicum (+26% in fruit weight), maize (+8% in root area), Lamb’s lettuce (+16% in dry weight, +39% in fresh weight), and strawberry (+14% in final crop yield), as well as improving the productivity of diary, animal, poultry, and aquaculture farming [28]. When in contact with water, Aquapulse^®^ is designed to generate an abundance of stable negatively charged nano- and micro-scale bubbles which can interact with positively charged particles in water to change its physico-chemical properties and the nature of its interactions with plants and soils [28].

In this study, we explore the use of PAW and Aquapulse^®^ feldspar-enriched water (APW), applied at different stages of the plant lifecycle, from seed germination to pre-harvest irrigation, with the aim of improving germination success and plant growth of *B. oleracea* and *M. sativa* grown in a soil based on simulated Martian regolith. APW, which is created by immersing a proprietary combination of feldspar minerals into water to introduce beneficial macro- and micro-nutrients and stable negatively charged nano- and micro-sized bubbles, differs from PAW in its mechanism of action: while PAW contains high concentrations of reactive oxygen and nitrogen species (e.g., NO_2_^−^, NO_3_^−^, H_2_O_2_) generated by the plasma treatment, APW does not contain these unstable oxidants or reactive species. Rather, irrigation with APW enhances plant growth by delivering additional essential minerals such as potassium, calcium, and sodium to the plant, with micro-bubbles stabilising nutrients and promoting their delivery, as well as increasing root-zone oxygenation. Given the differences in the mechanisms of action, we hypothesise that a combination of PAW and APW applied at different stages of plant development may improve seed germination success and crop performance.

## 2. Results and Discussion

This study investigates changes in germination success and plant growth in response to changes in the physico-chemical properties of water used for seed pre-soaking and plant irrigation for crops grown in soil based on simulated Martian regolith. As previously noted, the chemical profile of Martian regolith differs significantly from that of the terrestrial soil, with previous studies reporting poor seed germination success and stunted plant development [29]. In terrestrial agriculture practices, poor soils are often augmented with additives that e.g., improve their physical properties to ensure an appropriate level of movement of water and air, provide macro- and micro-nutrients that support plant development and growth, or deliver biochemically active substances e.g., signalling molecules capable of inducing a specific biological response in plants. The selection of these additives and methods for their delivery is typically constrained by their cost and energy budget, their abundance, and the environmental impact associated with their production and deployment, to name a few. In extraterrestrial agriculture, such as that on Mars, there may be additional constraints that would prioritise the selection of strategies that maximise the use of locally available resources, such as using local feldspar as a source of potassium and calcium [30], and multi-functional systems, such as plasma-based systems [31], that could be used for the generation of plant-stimulating reactive oxygen and nitrogen species, as well as for medical applications [32,33] and waste reforming [34,35]. Furthermore, as growth systems considered for Mars agriculture may prefer a closed-loop architecture, using irrigation water as a medium for the delivery of locally extracted mineral nutrients (i.e., in the form of APW) and signalling molecules (i.e., in the form of PAW) may offer an additional degree of efficiency, convenience, and control.

### 2.1. Water Chemistry

Table 1 summarises key physico-chemical properties of the three water types used in this study. From examination of this table, it is evident that, prior to any modification, normal water (NW) is slightly acidic, with a pH of 6.9 and a dissolved oxygen concentration of 2.3 mg/L. Its ammonia content and conductivity are below the detection limit of the commercial sensor used in this study.

In the case of PAW, upon plasma treatment, the pH of the water is reduced to 6.0, which is consistent with previously published reports [36,37], while the conductivity and ammonia levels remained below the detection limits for the instrument, and the level of dissolved oxygen is reduced to 0.3 mg/L. Plasma devices have previously been used to facilitate the conversion of atmospheric nitrogen into ammonia, and the use of nitrogen plasma in this study was driven in part by the goal of introducing bioavailable nitrates and ammonia into water [38,39,40,41] to mitigate the nitrogen deficiency in Martian regolith. It is also typical for plasma treatment to increase the conductivity of the treated water (often to within the µS/cm^−1^ range), as charge carriers are formed in the water, including, but not limited to, the generation of H_3_O^+^ and OH that results from interfacial H_2_O molecules being bombarded by plasma-generated N_2_^+^, N^+^, and O^+^ or the formation of H^+^ cations and NO_2_^−^ and NO_3_^−^ ions when HNO_2_ and HNO_3_ formed when plasma-generated RONS (e.g., OH) react with H_2_O dissolve. However, under the experimental conditions used in this study, plasma treatment did not yield a change in conductivity in the mS/cm^−1^ range, despite the increase in the concentration of H^+^ ions evident from the reduction in the pH by 0.9. Some of these reactions may have also consumed the dissolved oxygen present in the NW prior to plasma treatment.

In this study, PAW was applied immediately after its generation to ensure maximum availability of the reactive species. At the point of application, the concentration of longer-lived species, e.g., H_2_O_2_ was 4.764 ppm, whereas the concentration of O_3_ was estimated to be 2.85 × 10^−7^ M for PAW generated after 30 min of plasma treatment. It is worth noting that the chemistry of the plasma gas phase, and consequently the chemistry of PAW, is highly dependent on the combination of processing parameters used for plasma generation in combination with the nature of the systems used for treatment delivery [42]. From the perspective of agriculture, highly reactive species such as hydroxyl radicals (OH·), hydrogen radicals (H·), nitric oxide (NO), hydrogen peroxide (H_2_O_2_), nitrate (NO_3_^−^), nitrite (NO_2_^−^), ammonia (NH_3_), and ammonium ions (NH_4_^+^) are typically considered to be capable of influencing plant development when used for seed soaking (to induce germination and promote strong seedling emergence and early development of shoot and root systems) and for irrigation (to encourage biomass accumulation and promote stress and disease tolerance) [43,44]. The generation and accumulation of these species follows a series of chemical reactions that take place at the interface and in liquid bulk [42,45,46]. Electron impact reactions can lead to the breakdown of H_2_O and the formation of H· and hydroxyl (OH·) radicals [47,48], which are short-lived and highly chemically reactive [49]. Atomic N can then react with the hydroxyl radicals to produce nitric oxide, NO, which, in turn, can react with water and oxygen to yield nitric acid, HNO_3_, and nitrate ions, NO_3_^−^. Atomic N can also combine with hydrogen resulting from the dissociation of water to form ammonia (NH_3_) and ammonium ions (NH_4_^+^) [42].

In the case of APW, as a result of partial dissolution of some of the minerals and heterogenous bubble nucleation in the undulations on the feldspar surface, thus-prepared APW is enriched with minerals known to promote plant development (macronutrients such as K and micronutrients such as Ca and Mg) and an abundance of stable negatively charged nano- and micro-scale bubbles. These bubbles play an important role in nutrient delivery (by interacting with positively charged particles in water to change its physico-chemical properties) and micro-bubble-induced signalling. Figure S2 shows the size distribution of stable bubbles that are generated in APW after ~20 h of immersion of the Aquapulse^®^ feldspar cartridge in water. Measured by both high-speed video imaging paired with automated image analysis for optical detection and an ABS Acoustic Bubble Spectrometer^®^ for acoustic measurement, the size of the bubbles is estimated to fall within the 2 to 6 µm range. Due to their small size, these bubbles remain suspended in water rather than rising immediately to the surface. Over time they shrink and collapse, releasing their gas payload efficiently into the water [50]. The negatively charged bubbles also bind to and stabilise positively charged mineral ions in colloidal form, reducing leaching and maximising nutrient uptake, and mild reactive oxygen species signals at the bubble–water interface upregulate antioxidant defence and growth-promoting hormones, enhancing seed germination, root proliferation, and flowering and delivering measurable yield and quality gains across a range of crops [28].

When compared to NW, the alkalinity of APW increased, reaching a pH of 7.7. The dissolved oxygen concentration slightly increased from that of NW, attributed to the presence of gas-carrying bubbles; however, the level of ammonia remained below the detections limits of the commercial sensor used in this work. In contrast, at 55 mS/cm, the conductivity of APW was significantly higher than that of NW and PAW, suggesting a substantial increase in the number of charge carriers, which is likely due to the presence of the negatively charged bubbles and dissolved minerals. As previously noted, feldspars contain aluminium, silicone, and oxygen, along with other elements such as potassium, phosphorus, sodium, and calcium [30,51], with the most common feldspar minerals being albite (NaAlSi_3_O_8_), microcline (KAlSi_3_O_8_), and anorthite (CaAl_2_Si_2_O_8_) [52]. In the natural environment, feldspar minerals are stable in geological environments but are susceptible to decomposition by weathering in surface environments, at which point they release macronutrients such as potassium and calcium, along with soluble silicon in the form of silicic acid (H_4_SiO_4_) into the soil, with the reactions described by Equations (1)–(3) [53].

2NaAlSi_3_O_8_ + 2H^+^ + 9H_2_O ↔ Al_2_Si_2_O_5_(OH)_4_ + 4H_4_SiO_4_ + 2Na^+^ (1)

CaAl_2_Si_2_O_8_ + 8H_2_O ↔ 2Al(OH)_3_ + 2H_4_SiO_4_ + Ca^2+^ (2)

2KAlSi_3_O_8_ + 2H^+^ + 9H_2_O ↔ Al_2_Si_2_O_5_(OH)_4_ + 4H_4_SiO_4_ + 2K^+^ (3)

The reaction between the dissolved feldspar and H^+^ and OH^−^ ions will result in the formation of clay precipitates and the generation of new ions in the solution, the chemistry of which would depend on the nature of the original feldspar mineral [51]. From an agricultural perspective, both the formation of clays and the release of nutrients in the form of Na^+^, Ca^2+^, and K^+^ ions play an important role in improving soil quality and stimulating plant growth, with potassium required for activation of enzymes, photosynthesis, protein synthesis, and stomatal control of water use [54,55], calcium needed for cell wall structure development and intracellular signal transduction pathways [56], and silicon, though not yet widely recognised as an essential nutrient, demonstrated to enhance plant resistance to environmental stresses such as drought, pathogens, and salinity [57]. The negatively charged micro-bubbles in APW electrostatically attract positively charged mineral ions (e.g., Na^+^, K^+^, Mg^2+^) and then transport them to the root surface, where their concentrated presence enhances nutrient uptake and stimulates plant growth.

The elemental content, which was detected through ICP-MS, of the three forms of water (PAW, APW, and NW) shows different elemental level changes following plasma activation and mineral leaching treatment (Table 1). All were analysed through ICP-MS and expressed in parts per billion (ppb), as shown in Table 1.

In terms of elemental composition, NW contained Mg at a concentration of ~2.7 ppm, with Al (27.99 ppb), Fe (6.50 ppb), Cu (35.2 ppb), and Zn (11.77 ppb) also present at concentrations which fall within the typical value ranges for tap water. The level of P (at 725.43 ppb) was above that expected for natural background levels of phosphorus and can potentially be attributed to phosphate being added to water to prevent the release of e.g., Cu from the alloys used in plumbing.

The elemental composition of PAW was broadly similar to that of NW, with all elements well within the concentration ranges typically reported for tap water. This suggests that plasma treatment can be effectively used to introduce biochemically reactive RONS into water without significantly affecting its mineral profile. The levels of Mg (1.055 ppm), Mn (2.35 ppb), Fe (2.60 ppb), and Co (0.047 ppb) were most significantly reduced by plasma treatment, potentially through the reactions of these ions with plasma-generated RONS in water, which led to the formation of precipitates [58]. In contrast, the level of P, Ca, and K was higher in PAW, at 864.67 ppb, when compared to NW. Due to their high affinity for phosphates, iron and manganese readily bind to phosphates, limiting their free availability in water. Plasma treatment may facilitate the dissolution of these compounds, thereby increasing the availability of P.

In the case of APW, the dissolution and leaching of elements from aluminosilicates resulted in an increase in Al (33.51 ppb) and P (1036.66 ppb) levels compared to NW. The presence of P may be due to dissolution of the feldspar component apatite [(Ca_5_(PO_4_)_3_ (F, OH, Cl)], which, though it is not common in feldspar, can be associated with feldspar in rocks. Higher levels of Fe (5.79 ppb), Mn (3.29 ppb), Cu (60.11 ppb), Mg (3.5 ppm), Ca (12.5 ppb), and K (49.8 ppb) were present in APW compared to NW, indicating effective mobilisation of micronutrients from the APW. A detailed examination of the elemental composition of individual feldspar stones was performed by ICP-OES, with the results reported in the Supplementary Information (Table S1).

### 2.2. Crop Productivity Under Different Soaking and Irrigation Regimens

The effect of exposing seeds and plants to water enriched with either biochemically active RONS generated by atmospheric pressure plasma (PAW) or enriched with macronutrients, micronutrients, and charged nano- and micro-scale bubbles generated by the dissolution of feldspar (APW) was then investigated for two plant species, *M. sativa* and *B. oleracea*. The plants were subjected to a total of nine regimens of soaking + irrigation, namely NW soaking + NW irrigation (control), NW soaking + APW irrigation, NW soaking + PAW irrigation, APW soaking + NW irrigation, APW soaking + APW irrigation, APW soaking + PAW irrigation, PAW soaking + NW irrigation, PAW soaking + APW irrigation, and PAW soaking + PAW irrigation, to understand if such supplementation is beneficial at different stages of the crop lifecycle.

**Figure 1:**
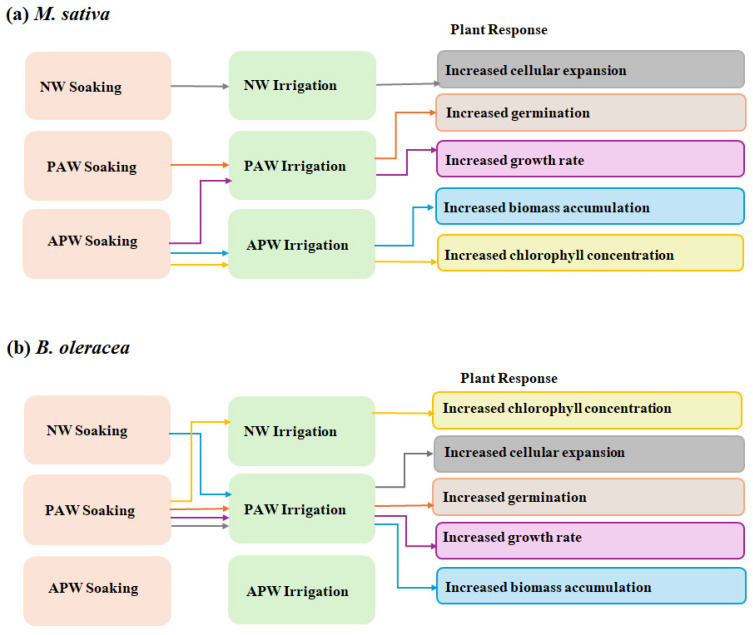
Comparative beneficial effects of seed soaking + irrigation regimens on biomass and photosynthetic performance in (**a**) *M. sativa* and (**b**) *B. oleracea* grown in a soil based on simulated Martian regolith. The arrows in the figure represent sequence of application and representative plant response.

Figure 1 highlights a select number of examples of the beneficial effects that specific soaking and irrigation regimens have on seed germination and subsequent plant development in *M. sativa* and *B. oleracea*, suggesting the important role the optimisation of soaking and irrigation regimens may play in optimising the performance of crops that may have different growth needs.

In this work, the selection of plant species was informed by their relatively high tolerance to such environmental conditions as high salinity, different nutrient requirements, and their suitability to be grown as microgreens with high nutritional value and a diverse flavour profile [59,60,61,62,63]. Briefly, *M. sativa* is a highly adaptable perennial legume known for its exceptional agronomic and nutritional value which can be grown productively in arid and semi-arid conditions, with a preference for deep, well-drained, neutral-pH soils [64]. The nitrogen-fixing quality of *M. sativa* reduce its dependence on chemical fertilisers and enhances the fertility of soils on which the crops are cultivated [65]. This makes *M. sativa* a potentially promising candidate for Martian agriculture, where regolith is deficient in both organic matter and nitrogen content, with the possibility of exploiting legume–rhizobia symbiosis for rhizobia-mediated fixation of the nitrogen that is present in small quantities in the Martian atmosphere. Previous results have demonstrated root nodules developing on *Medicago truncatula* roots when grown in the presence of its nitrogen-fixing symbiotic partners, *Sinorhizobium meliloti* and *Sinorhizobium medicae*, on different grades of Mojave Mars Simulant (MMS-1 and MMS-2) [12]. Similar results were found for a *Melilotus officinalis*/*Sinorhizobium meliloti* symbiotic partnership grown on MMS-1 regolith [13], with NH_4_ production primarily limited to plant uptake and insignificant deposition of excess plant-available N into the regolith growth medium via rhizodeposition of exudates. Additionally, *M. sativa* has high biomass output and rich protein content [66], and thus can potentially serve both as a nutritional food crop (e.g., microgreens) and an organic fertiliser (e.g., compost or green manure), further enhancing Mars’ sustainable food system.

*B. oleracea* prefers an acidic environment with a pH range of 5.5 to 6.5, as this allows for the full development and stability of its natural pigments (anthocyanins) [67], which are known to have health benefits, and a high nutritional content. *B. oleracea* is rich in vitamins A, C, E, K, and B-complex (B1, B2, folate), as well as minerals such as calcium, iron, magnesium, and potassium. It also contains powerful antioxidants, phytochemicals, and secondary metabolites, such as glucosinolates and flavonoids, that possess numerous health benefits such as anti-cancer, anti-inflammatory, and immune-stimulatory activities. Under more alkaline conditions, these pigments begin to break down, and the loss of pigments and other nutrients occurs [67]. As a crop, *B. oleracea* is adaptable, with high yield density, making it a potentially strategic crop for Martian agricultural systems [68].

#### 2.2.1. Biomass Accumulation

**Figure 2:**
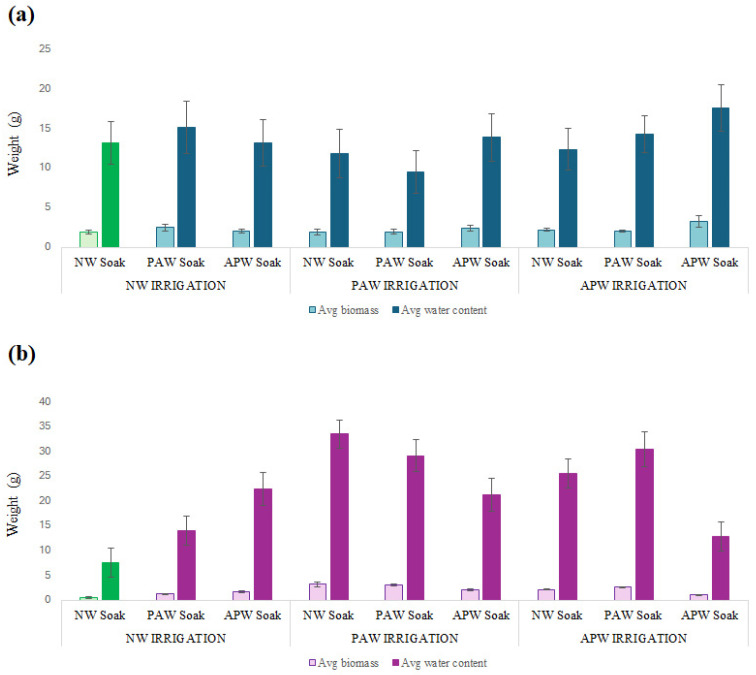
Total wet weight and dry weight of biomass harvested from (**a**) *M. sativa* and (**b**) *B. oleracea* after 40 Martian days (sols) of cultivation in soil based on simulated Martian regolith, as a function of nine different soaking and irrigation regimens. Values are presented as mean ± SD. Green colour represent control.

As is evident from Figure 2 and Table 2, the combination of initial mineral enrichment during APW soaking followed by sustained mineral supplementation with APW irrigation produced the best general performance for *M. sativa*, enhancing biomass by 67% and water content by ~33% when compared to plants from the NW soaking + NW irrigation control group. Micro-bubble irrigation is known to increase dissolved oxygen availability in the root zone, which, in turn, increases the respiration of roots and the energy supply for nutrient uptake, while simultaneously increasing the solubilisation and mobilisation of key mineral nutrients (N, P, K, and micronutrients) [69]. This also helps the growth of beneficial microbial populations (e.g., rhizobia and mycorrhizae) that facilitate nitrogen fixation and phosphorus acquisition. Sufficient nitrogen from symbiotic rhizobia, supplemented by improved oxygenation and phosphorus availability, enhances N-fixation. Increased phosphorus supports energy transfer, while adequate potassium regulates stomata, activates enzymes, and maintains osmotic balance. Together, these factors maximise photosynthetic capacity and resource allocation to growth, resulting in elevated fresh and dry biomass [70,71]. Conversely, both biomass accumulation and water retention were most significantly reduced when plants were subjected to oxidative stress conditions, both in the case of PAW soaking + PAW irrigation and NW soaking + PAW irrigation. While the impact on biomass accumulation was moderate, at approximately +0.7% and –3.5%, respectively, the reduction in water retention was notably more pronounced, at –28% and –10.2%, respectively. Excessive ROS/RNS exposure may lead to excessive membrane disruption, lipid peroxidation, and stomatal malfunction [72]. Interestingly, the presence of nano-/micro-bubbles and mineral priming in the form of APW soaking can help plants mitigate later oxidative stress during PAW irrigation, with an overall increase in biomass of +22.7% and in water content of +5.1% when compared to the control. Similarly, mineral supplementation in the form of APW irrigation can minimise the effects of oxidative stress during PAW soaking, resulting in modest increases in the biomass and water contents of 7.0% and 8.2%, respectively. These observations suggest the importance of controlling oxidative stress, along with nutritional supply, in a way that promotes development and physiological health in plants.

In contrast to *M. sativa*, the impact of RONS stimulation and mineral supplementation was significantly greater for *B. oleracea*, which demonstrated a biomass increase of approximately 477% and a water content increase of approximately 343% in the case of the NW soaking + PAW irrigation when compared to the control. Other treatment regimens involving PAW also produced strong enhancements in both biomass and water content, suggesting both oxidative priming and sustained delivery of ROS/RNS as promising strategies to stimulate plant growth, enhance nutrient uptake, and promote physiological processes such as osmotic regulation and stomatal function in plants with similar needs to *B. oleracea* [73]. Recent studies have shown that PAW irrigation could cause overexpression of transmembrane transport, cell wall biogenesis, and oxidoreductase activity genes, leading to increased biomass and improved water status in plants such as cabbage and sorghum [73,74]. In addition, the nitrate content in PAW delivers a direct form of nitrogen, promoting plant growth and crop production [75].

The APW soaking + APW irrigation regimen delivered the lowest level of increase, at +96% for biomass and +70% for water content when compared to control, suggesting that introducing mineral supplementation during both the soaking and irrigation stages may be counterproductive, potentially leading to ionic interference, osmotic imbalances, or precipitation of nutrients that may limit nutrient bioavailability and hinder maximum water uptake. Excessive or unbalanced mineral exposure may reduce nutrient assimilation efficiency and potentially induce physiological stress. This is supported by a much greater improvement in biomass accumulation and water content for APW soaking + NW irrigation (at +216.7% and +196.1%, respectively) and NW soaking + APW irrigation (at +294.7% and +238.2%, respectively).

These contrasting outcomes indicate that, in *B. oleracea*, controlled oxidative signalling through PAW irrigation will significantly improve plant functioning through stimulation of growth and defence processes, and excessive or unbalanced mineral supplements will result in a smaller level of improvement.

#### 2.2.2. Seed Germination

**Figure 3:**
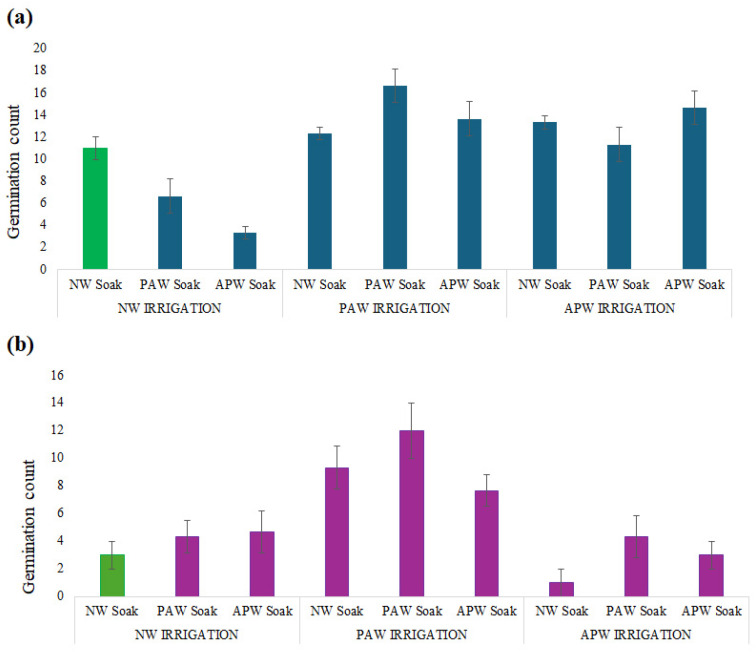
Germination counts in numbers for (**a**) *M. sativa* and (**b**) *B. oleracea* after 14 sols in soil based on simulated Martian regolith, shown for nine different soaking and irrigation regimens (NW, APW, or PAW). For each treatment, 120 seeds of each species were sown at a depth of 3 mm and monitored over 14 Martian sols under controlled conditions (light intensity 590 µmol m^−2^ s^−1^; temperature cycle 20 °C/12 °C). Values are presented as mean ± SD. Green colour represent control.

From examination of the results in Figure 3 and Table 3, it is evident that *M. sativa* and *B. oleracea* have different tolerances and responses to exposure to specific levels of ionic and RONS species. In *B. oleracea*, a leafy non-legume with a high growth rate, the seed coats were harder when compared to *M. sativa*. As such, they may have benefited to a greater degree from the exposure to more acidic, RONS-rich PAW during both soaking and irrigation, as oxidative stimulation with RONS is known to activate cellular metabolism, increase membrane permeability, and induce antioxidant defence mechanisms in plants [16,21]. RONS such as H_2_O_2_ and NO have been shown to induce rapid germination through the disruption of ABA pathways and the stimulation of GA signalling, as well as by changing the redox status, loosening the cell wall, and promoting radicle protrusion. Indeed, exposure to PAW has been shown to increase the germination rate of seeds, water uptake, seed vigour, and seedling growth in an extensive variety of crops, encompassing mung bean, wheat, spinach, soybean, and tomato [76]. These improvements are due partly to the ability of RONS to alter seed surface properties, improving wettability and water absorption, and activate antioxidant enzymes and the expression of beneficial genes (e.g., TOR, GRF, HSPs) [19]. Additionally, exposure to PAW has also been proven to enhance germination and stem elongation, generally by generating NO and acidifying the solution [77,78]. Cumulatively, pre-germinative metabolic priming and post-sowing RONS stimulation could enhance germination success and seedling emergence under stresses associated with cultivation under Martian conditions.

In contrast, the regimen of NW soaking followed by APW irrigation resulted in the lowest germination success for *B. oleracea*, potentially attributed to the metal-induced suppression of nutrient mobilisation and embryo development. The same mechanism was also observed in another study, where treatment with 5 mmol L^−1^ copper reduced germination by 37% and embryonic axis growth by 70% in *Phaseolus vulgaris* L. [79]. In APW, the levels of Cu (at 60.11 ppb) exceed those in PAW and NW and may be sufficient to interfere with seed hydration in species with lower metal tolerance, such as *B. oleracea* [80]. Moreover, while moderate concentrations of nano-/micro-bubbles can enhance seed germination, high levels of bubbles can suppress germination due to excessive oxidative stress [81].

For *M. sativa*, the best treatment outcome was observed when PAW was used for both soaking and irrigation, followed by APW use for both soaking and irrigation and APW soaking and PAW irrigation. This shows that *M. sativa* was positively responding to sustained oxidative signalling, providing evidence of the potential efficacy of sustained oxidative support for seedling establishment. These results also suggest that *M. sativa* benefited from mineral supplementation and from the presence of nano-/micro-bubbles when exposure to APW was sustained. The beneficial effect of double APW treatments in *M. sativa* contrasts with the response of *B. oleracea*, indicating species-specific levels of ion tolerance. *M. sativa* may have more efficient ion regulatory mechanisms, such that it can capitalise on nutrient availability without compromising osmotic balance. Conversely, the lowest level of germination success for *M. sativa* was observed when APW soaking was followed by NW irrigation. The mineral exposure during priming may have imposed cellular stress, and in the absence of follow-up RONS signalling or ionic moderation, seed hydration and metabolic recovery may have been impaired.

Overall, both species showed improvement with PAW irrigation, regardless of the type of water used for seed soaking, suggesting that oxidative stimulation during very early stages of seed germination and growth is a broadly beneficial mechanism, perhaps because of its role in RONS-mediated signalling, activation of antioxidant enzymes, and optimisation of water uptake dynamics [82].

#### 2.2.3. Shoot Development

**Figure 4:**
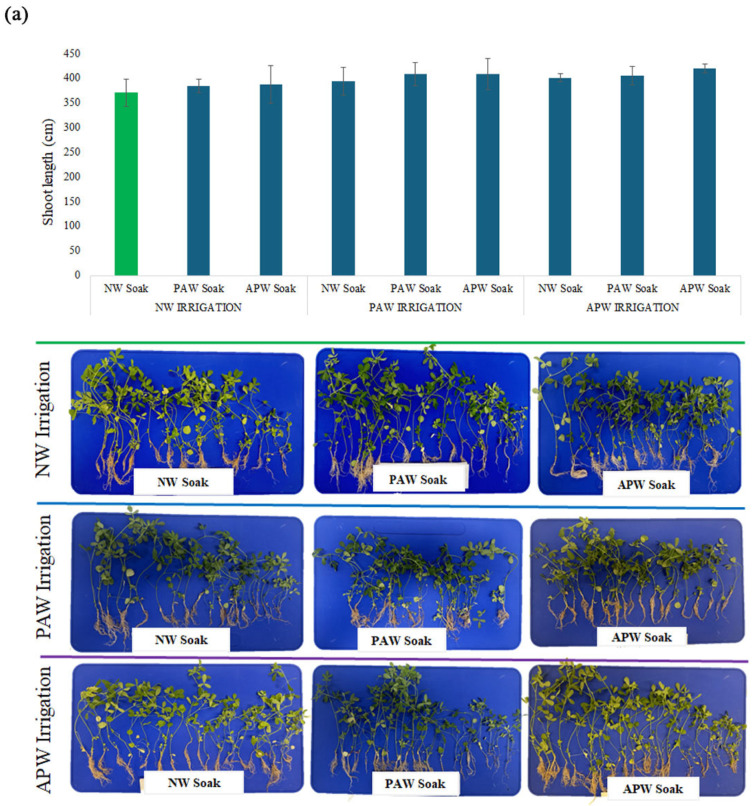

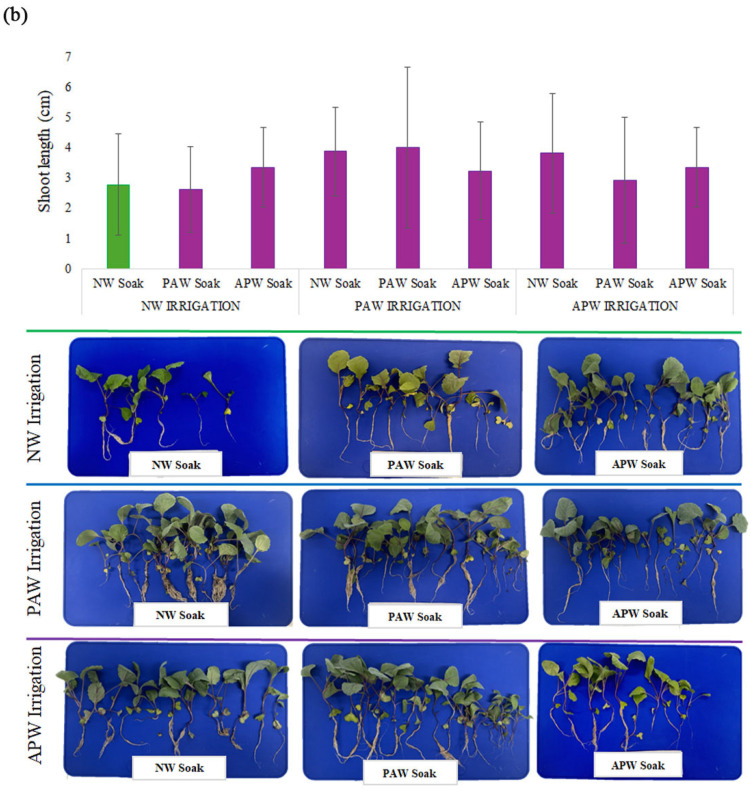
Average shoot length for (**a**) *M. sativa* and (**b**) *B. oleracea* after 40 sols of cultivation in soil based on simulated Martian regolith, as a function of nine different soaking and irrigation regimens. The measurements are conducted on freshly harvested crops. Values are presented as mean ± SD. Green colour represent control.

As is evident from examination of the shoot length results summarised in Figure 4 and Table 4, the impact of the choice of the soaking and irrigation regimen was significantly greater in the case of *B. oleracea* when compared to *M. sativa*, highlighting once again the differences in the needs of different crops at different stages of their development. In the case of *M. sativa*, the chemistry of water used for soaking and irrigation played a very minor role in determining the shoot length, with the highest value of the average shoot length produced in plants subjected to APW soaking followed by PAW irrigation, at 17.9 ± 4.4 cm. This was only marginally higher (+3.8%) than the average shoot length in plants from the control group using NW for both soaking and irrigation, at 17.3 ± 5.1 cm. Broadly, exposure to nano-/micro-bubbles would have elevated plant levels of gibberellins, which are directly responsible for increased plant nutrient absorption and stem elongation [83]. Subsequent sustained exposure to RONS may also have promoted shoot elongation, suggesting a conditioning synergy between oxidative stress and the presence of nano-/micro-bubbles.

*M. sativa* is a legume with a high demand for mineral nutrients such as potassium and magnesium that could be made available through exposure to mineral-rich APW. If potassium ions are present in the water, even at very low concentrations, increased water uptake could lead to higher overall absorption of potassium by the plant over time. Potassium is important in promoting successful seed growth, as it supports various physiological and biochemical processes, including the stimulation of energy metabolism; potassium activates key enzymes such as ATP synthase and H^+^-ATPase with functions in the synthesis of ATP and membrane transport in the developing seedling early in life. In addition, potassium helps modulate water uptake and osmotic balance, both of which are critical during the rehydration stage of germination [84]. By modulating the turgor pressure of cells, potassium enables cell expansion and radicle emergence. Germination triggers a surge of metabolic activity that may generate reactive oxygen species (ROS), and potassium mitigates such oxidative stress by suppressing NADPH oxidase activity and enhancing the detoxifying activity of antioxidant enzymes. Furthermore, potassium is linked directly to hormone signal transduction pathways; it influences the activity of hormones such as auxins, abscisic acid (ABA), gibberellins (GA), and cytokinins, which are responsible for the regulation of dormancy, enzyme activation, and cell elongation in seed germination. Lastly, potassium enhances the tolerance of the seed to environmental stresses such as drought or temperature changes, by membrane stabilisation and physiological flexibility [84]. Similarly, magnesium is required by leguminous plants during the early stage of development, as it helps with effective water and nutrient uptake [85]. Mg is involved in energy metabolism, photosynthesis, protein synthesis, carbon partitioning, and stress tolerance in plants. Mg is also vital in nitrogen metabolism and transport, affecting nodule development by controlling carbon–nitrogen movement and changing the plasmodesmata permeability of nodule cells. These activities combined enhance plant growth and seed protein content in legumes [85].

Considering that there was only a modest improvement in shoot length as a result of APW exposure, there appears to be strong potential to further enhance the response by introducing a greater concentration of minerals such as K, Ca, and Mg into APW by e.g., extending the duration of the leaching process or introducing heating or agitation to promote feldspar dissolution. Post-sowing, PAW irrigation may benefit shoot elongation by triggering mild oxidative stress by ROS/RNS species such as H_2_O_2_, NO_3_^−^, and NO_2_^−^ [21,44]. As previously noted, exposure to RONS increases water absorptivity and breaks seed dormancy, while also killing pathogenic microorganisms that may compromise seed germination or subsequent seedling growth [86,87,88]. However, the response to RONS is dependent on the dose, as well as on the stage of plant development when the treatment is applied and the environmental conditions and treatments the plants are subsequently subjected to. This is exemplified by the lower shoot length, at 15.2 ± 5.5 cm, corresponding to an −11.8% decrease in comparison with control, observed in plants subjected to PAW soaking followed by APW irrigation. Here, there may have been antagonistic effects between the initial oxidative priming that stimulated metabolism and enzyme activity and the sustained mineral supplementation during APW irrigation. The ionic composition of APW, particularly the higher concentrations of elements like copper or manganese, could have created unfavourable osmotic or redox imbalances during later stages of growth.

In contrast, *B. oleracea* was significantly more sensitive to several of the watering regimens used in this study, with particularly favourable results obtained for the combination of PAW soaking and PAW irrigation, likely due to oxidative priming and sustained RONS stimulation (by way of H_2_O_2_, NO_3_^−^, and NO_2_^−^ species) that facilitated metabolic enzyme activation, leading to the promotion of germination and quick cell growth. Under this treatment regime, *B. oleracea* plants with an average shoot length of 4.0 ± 2.6 cm were obtained, which was 44% longer than that for the plants in the control group receiving NW for soaking and irrigation. Reactive species, including hydrogen peroxide (H_2_O_2_), nitric oxide (NO), and nitrates/nitrites, have been demonstrated to act as signalling molecules that enhance physiological responses in plants during their early development [77,89]. In *B. oleracea*, a species naturally adapted to thrive in mildly acidic conditions, the acidic nature of PAW and the presence of RONS, which trigger a mild stress response, may stimulate shoot elongation, induced nutrient uptake through root zone acidification, and enhanced antioxidant enzyme activities. The double exposure to PAW likely provided a prolonged biochemical and pH environment favourable for shoot growth.

In contrast, PAW soaking followed by NW irrigation resulted in a shorter average shoot length, at 2.6 ± 1.4 cm, which corresponded to a 6% decrease when compared to the control. Despite the initial priming with PAW, the switch to normal water for irrigation would have caused the levels of beneficial reactive species to decrease, potentially creating an imbalance in redox signalling, with an increase in pH potentially negatively impacting root ion exchange and solubilisation of nutrients, which are particularly important for a pH-sensitive crop such as *B. oleracea.*

#### 2.2.4. Leaf Architecture

**Figure 5:**
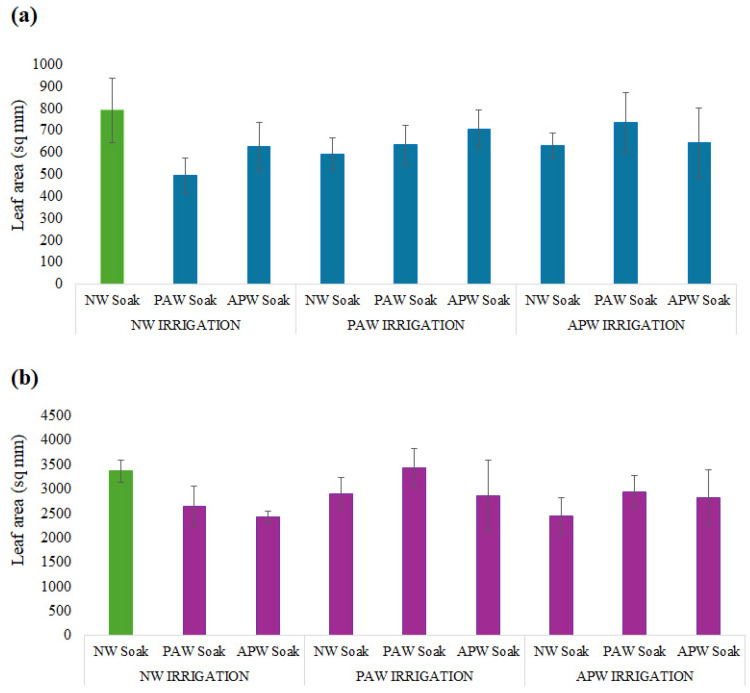
Average leaf area of (**a**) *M. sativa* and (**b**) *B. oleracea* after 40 sols of cultivation in soil based on simulated Martian regolith, as a function of nine different soaking and irrigation regimens. Values are presented as mean ± SD. Green colour represent control.

Examination of the average leaf area, as presented in Figure 5 and Table 5, reveals that soaking of seeds and subsequent irrigation with NW results in plants with the greatest average leaf area: 791 ± 147 mm^2^ in the case of *M. sativa*. The quality of tap water used in this study is very high, with very low levels of potentially toxic metals. Furthermore, it had the highest level of Mg of the three types of water used in this study. Among the remaining soaking and irrigation regimens, the reduction in leaf area was the least significant for the combination of PAW soaking + APW irrigation (−7.2%) and APW soaking + PAW irrigation (−11%), suggesting that there may be antagonistic effects between changes induced by RONS exposure and those induced by the presence of greater levels of certain minerals.

For *B. oleracea*, a regimen where PAW was used for both soaking and irrigation produced plants with an average leaf area similar to that in the control group, at 3440 ± 392 mm^2^, corresponding to a slight increase of 2% compared to the NW control, at 3368 ± 232 mm^2^. As previously noted, exposure of seeds to PAW via soaking can break seed dormancy, enhance germination success, and stimulate cotyledons. Post-germination, ROS/RNS in PAW irrigation can promote cell expansion and nutrient uptake and possibly trigger signalling pathways and metabolic processes [89] associated with leaf growth and development [90]. PAW also has the capacity to enhance nutrient supply and uptake by altering the physico-chemical characteristics of soil, resulting in a greater leaf area [90]. Interestingly, when PAW was used in combination with NW, either as PAW soaking + NW irrigation (−21%) or NW soaking + PAW irrigation (−13%), there was a reduction in the average leaf area when compared to the control group. This suggests that, where the use of NW for both soaking and irrigation delivers a stable and consistent environment, switching between NW and PAW may potentially introduce a degree of stress requiring mobilisation of plant resources and an adaptation period for the plant to transition from one type of water to another.

The reduction in the average leaf area was particularly significant for watering regimens employing APW, at ~−27% for crops subjected to APW soaking + NW irrigation, APW soaking + PAW irrigation, and NW soaking + APW irrigation. In addition to being more alkaline when compared to NW and PAW, APW also has a significantly greater concentration of ions, which may introduce osmotic stress or ion toxicity, which in turn may prevent initial tissue development and turgor-enhanced cell growth. Interestingly, when seeds are subjected to PAW soaking prior to APW irrigation, the reduction in the average leaf area is less pronounced, suggesting that oxidative priming may activate defence mechanisms that would make the seedlings more resilient and tolerant to the subsequent ionic stress.

#### 2.2.5. Chlorophyll Content

**Figure 6:**
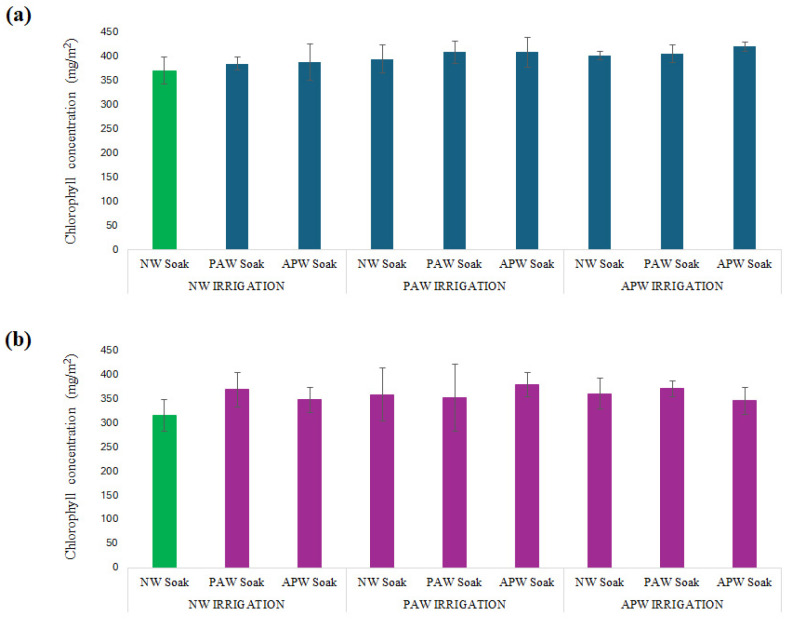
Chlorophyll concentration in (**a**) *M. sativa* and (**b**) *B. oleracea* after 40 sols of cultivation in soil based on simulated Martian regolith, as a function of nine different soaking and irrigation regimens. Values are presented as mean ± SD. Green colour represent control.

All treatment regimens considered in this study had a positive effect on chlorophyll accumulation in both *M. sativa* and *B. oleracea*, as shown in Figure 6 and Table 6: up to 13.2% for APW soaking + APW irrigation for *M. sativa* and up to 22% for PAW soaking + NW irrigation for *B. oleracea*. Broadly, the results suggest that neither PAW nor APW has an adverse impact on chlorophyll biosynthesis, suggesting that all the water types used in this study are compatible with the maintenance of photosynthetic efficiency, even when plants are grown in the challenging Martian regolith. In the case of *M. sativa*, ranging from 4.3% to 13.2% above control (371 mg/m^2^), the increase in chlorophyll concentration was consistently below that observed in *B. oleracea*, regardless of the regimen considered. The most effective regimen involved the use of APW for both soaking and irrigation, which elevated the chlorophyll concentration to 420 mg/m^2^ by supplying K^+^ and micronutrients that are cofactors in chlorophyll biosynthesis on a continuous basis [91,92]. The nano-/micro-bubbles present in APW can elevate the DO content in the root zone and thereby enhance ATP production for chlorophyll biosynthesis. This can also improve the availability and uptake of key mineral co-factors such as Mg^2+^ and Fe^2+^, notably increasing chlorophyll levels in plants grown in poorly aerated soil [93], such as Martian regolith. A comparatively lower increase in chlorophyll content in *M. sativa* was found with a regimen involving PAW soaking and NW irrigation (at 385 mg/m^2^, +3.8%), which is likely due to the transient nature of ROS/RNS priming. While PAW induces antioxidant and chlorophyll-gene expression upon soaking, an absence of persistent oxidative cues or additional mineral cofactors during plant development may lead to limited pigment synthesis [94,95].

For *B. oleracea*, most treatments produced plants with comparable contents of chlorophyll; however, the combination of PAW soaking and NW irrigation delivered crops with the highest chlorophyll content (385 mg/m^2^, +22.2%), demonstrating that ROS/RNS priming during the seed development stage significantly stimulates key enzymes in the tetrapyrrole pathway and enhances antioxidant defences [96]. The APW soaking + PAW irrigation (379 mg/m^2^, +20.3%) and PAW soaking + APW irrigation (370 mg/m^2^, +17.5%) treatments also showed similarly strong improvements, consistent with additive effects of either ROS/RNS signalling in the late stage or mineral supplements to stimulate chlorophyll production. In contrast, the use of APW for both soaking and irrigation was associated with a lesser increase in chlorophyll concentration (346 mg/m^2^, +9.8%), most likely due to the prolonged exposure of plants to water with high ionic strength, which may have interfered with enzyme activity and pigment stability.

The minimal difference in chlorophyll content also suggests that the differences in biomass or shoot growth reported here may not be regulated by photosynthetic pigment accumulation. Instead, such growth differences may be due to increased water uptake, nutrient assimilation, or early development of the root system, most significantly in PAW-treated plants [44,97]. The RONS present in PAW may have the capacity to modulate stress responses and enhance early metabolic processes [89], specifically in species such as *B. oleracea* with tougher seed coats.

#### 2.2.6. Vascular Architecture

**Figure 7:**
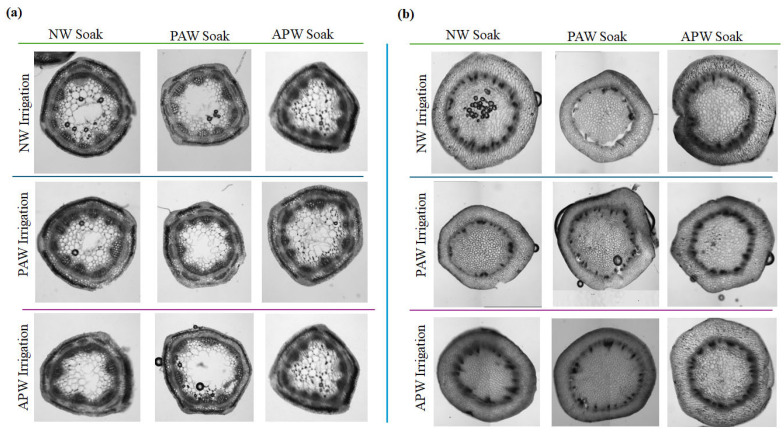
Stem cross-sections of (**a**) *M. sativa* and (**b**) *B. oleracea* after 10 sols of cultivation in soil based on simulated Martian regolith under nine different soaking and irrigation regimens. Sections were collected between the first and second stem nodes and imaged at 10× magnification (scale = 200 μm). Stem structural quality was ranked using a semi-quantitative integrity scoring system adapted from [100]: 0 = high integrity with symmetrical and uniform tissue distribution; 1 = slight asymmetry or minor irregularities; 2 = moderate asymmetry with uneven tissue distribution. *M. sativa* generally maintained high integrity (scores 0–1) across treatments, whereas *B. oleracea* displayed greater variation (scores 1–2), indicating species-specific responses to irrigation type and soaking regime.

Microscopic examination of stem cross-sections shows some anatomical differences in plants cultivated under different soaking and irrigation regimens (Figure 7). In plants exposed to PAW soaking, the cross-sections exhibit more clear vascular bundles and more developed xylem and phloem tissues, especially in *M. sativa*. This suggests that PAW soaking may promote vascular differentiation and enhance the transport of nutrients and water more efficiently [98,99], which is an advantage for plants grown in soils that have a dense, compacted structure and limited nutrient availability.

Cross-sections of *M. sativa* stems subjected to PAW soaking and irrigation had areas of dense xylem tissue with some disorganisation of cell walls, potentially suggestive of oxidative or osmotic stress from overexposure to RONS. Though APW and NW soaking in PAW irrigation maintained a higher percentage of healthy vascular structure, significant loss in central pith turgor did occur, especially for plants with seeds subjected to NW soaking.

In *M. sativa* exposed to mineral supplementation through APW irrigation, cross-sections suggested the presence of some unbroken vascular bundles and unbroken tissue organisation. Here, PAW soaking overall led to increased tissue tightness and tighter central pith, suggesting possible increased lignification or stress-mediated increased cell wall thickness. When compared to cross-sections of plants subjected to APW seed soaking that showed uniform vascular clarity and moderate cell wall thickening, in groups where NW soaking was used, cross-sections showed slightly reduced structural definition.

When irrigated with NW, the stems of *M. sativa* had consistent tissue integrity irrespective of the type of soaking, although some images suggest the possible presence of internal defects such as voids or cell disruption in plants grown from seeds subjected to PAW soaking. This can be an indication of a stress response or cell osmotic disruption. APW and NW soaking under NW irrigation appear to promote the preservation of vascular architecture, with cross-sections of plants subjected to APW soaking showing a potentially healthier architecture.

In *B. oleracea*, APW irrigation produced plants with round, turgid stem organs with well-defined vascular structure when soaked in APW. NW and PAW soaking under APW irrigation introduced slight irregularities, with plants where seeds were subjected to PAW soaking exhibiting an increased xylem area and less well defined boundaries. NW irrigation appeared to yield plants with the most consistent stem cellular morphology, regardless of the soaking used. The cellular structure of stems where seeds were subjected to PAW soaking were especially dense, with well-defined vascular rings, while soaking in NW and APW resulted in plants with uniform stem tissue patterns. In *B. oleracea* plants irrigated with PAW, the cellular structure appeared to be more compact, similar to what was observed in *M. sativa*.

The stem cross-sections of plants exposed to PAW seed soaking exhibited a vascular thickened area and an increase in tissue density, which could be a common physiological response to repeated RONS exposure. The stems of plants subjected to APW soaking followed by PAW irrigation maintained greater uniformity and vascular definition, suggesting a stabilising action of APW even under stress-inducing irrigation conditions. Generally, PAW soaking caused considerable anatomical changes in both species, particularly with regard to tissue compaction and potential lignification reactions. *M. sativa* demonstrated the greatest level of structural change as a result of exposure to PAW irrigation, particularly when combined with PAW soaking. By contrast, *B. oleracea* appeared to be less susceptible to the RONS-mediated effects of PAW irrigation, while PAW soaking enhanced vascular density. In both species, the stems of plants subjected to APW soaking maintained vascular integrity, indicating a potentially protective role of APW soaking against subsequent osmotic or oxidative stress.

## 3. Materials and Methods

### 3.1. Experimental Design

**Figure 8:**
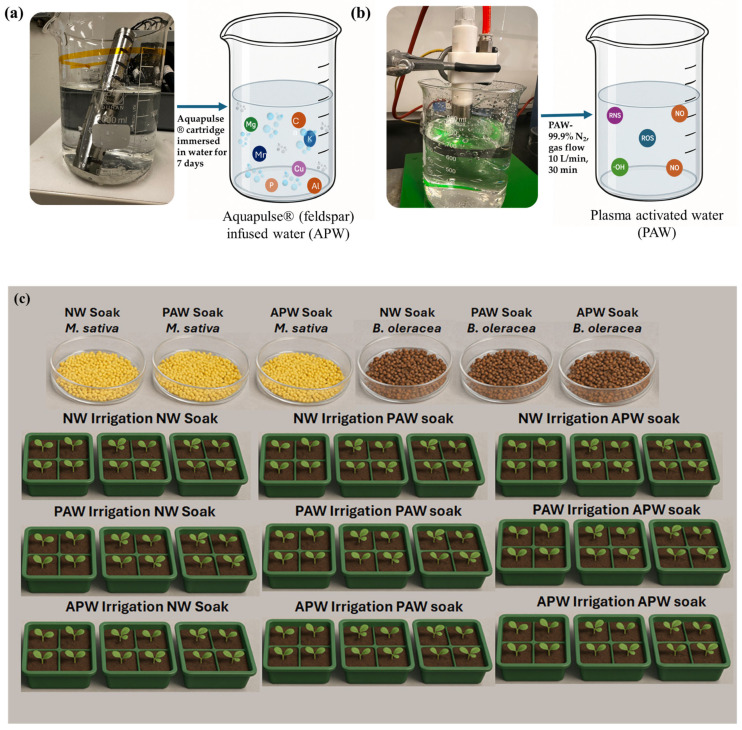
(**a**) Preparation of APW. A permeable Aquapulse^®^ stainless steel cartridge containing a proprietary combination of feldspar minerals is immersed in normal tap water. Dissolution-driven nano-/micro-bubble generation and leaching of macronutrients (e.g., potassium) and micronutrients (e.g., calcium) from feldspar into water occur over 7 days under ambient conditions. (**b**) Preparation of PAW. Water is treated with an atmospheric pressure nitrogen plasma jet for 30 min to introduce highly biochemically reactive oxygen and nitrogen species with the ability to significantly influence biological systems. (**c**) Images of seeds and plants at different stages of the germination and growth experiment. Top panel: Soaking of seeds of *B. oleracea* and *M. sativa* in NW, PAW, and APW prior to sowing. Bottom panels: Seedlings of *B. oleracea* and *M. sativa* grown under nine different pre-soaking + irrigation regimens in soil containing 10 parts simulated Martian regolith and 1 part coconut fibre.

The experimental design is summarised in Figure 8.

#### 3.1.1. Growth Medium

In this study, Exolith Lab^®^ Mars Global (MGS-1) High-Fidelity Martian Regolith Simulant was purchased from Space Resource Technologies, Oviedo, USA and used without further modification. MGS-1 is analogous to basaltic soils, the data for which was collected by the Mars Science Laboratory Curiosity rover. MGS-1 has an uncompressed bulk density of 1.40 g/cm^3^, a mean and median particle size of 63 μm, and a particle size range of <0.04–1000 μm. MGS-1 is produced by combining terrestrial anorthosite (27.1%), glass-rich basalt (22.9%), bronzite (20.3%), olivine (13.7%), magnesium sulphate (4.0%), ferrihydrite (3.5%), hydrated silica (3.0%), magnetite (1.9%), anhydrite (1.7%), ferrous carbonate (1.4%), and hematite (0.5%).

As it lacks organic matter, regolith was mixed with coconut fibre (Brunnings Garden Products Pty Ltd., Oakleigh South, Australia) at a ratio of coir to regolith of 1:10 by weight, with the resultant product referred to as ‘soil’ hereafter.

#### 3.1.2. Water for Soaking and Irrigation

In order to understand the effects of the physico-chemical properties of water on the development of plants grown on Martian-like soil, three types of water were used, referred to as NW, APW, and PAW. NW represents tap water that was used without further modification.

APW water was prepared by immersing feldspar particles contained within a stainless steel cartridge into 1000 mL of tap water in a beaker, allowing for leaching of the minerals and nano-/micro-bubble generation to take place over a period of 7 days under ambient conditions (Figure 8a). Feldspars are aluminosilicate minerals with varying amounts of potassium, sodium, and calcium in a solid solution series, and their composition can be expressed in terms of KAlSi_3_O_8_ orthoclase, NaAlSi_3_O_8_ albite, and CaAl_2_Si_2_O_8_ anorthite endmembers. Feldspars have been used in agriculture as a source of macronutrients such as potassium and micronutrients such as Ca and Mg, although the abundance of Al_2_O_3_ presents a challenge, especially where strategies for the accelerated release of nutrients are employed. In this study, Aquapulse^®^ cartridges containing a proprietary combination of different feldspar crystals were used.

**Figure 9:**
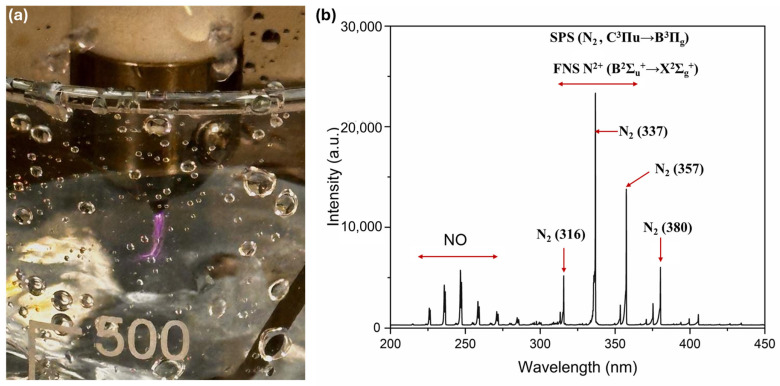
(**a**) Photograph of the cold atmospheric plasma plume generated in tap water using nitrogen gas. (**b**) Corresponding optical emission spectrum (200–450 nm) of the plasma plume in proximity to the plasma–liquid interaction zone. The spectrum features the N_2_ second positive system (SPS, C^3^Πᵤ → B^3^Πg) with strong peaks at 337 nm, 357 nm, and 380 nm, the N_2_^+^ first negative system (FNS, B^2^Σᵤ^+^ → X^2^Σg^+^) spanning 300–365 nm, and molecular NO emissions in the 220–260 nm region. Key peak assignments that are N_2_ (316 nm, 337 nm, 357 nm, 380 nm) are indicated by red arrows.

PAW was prepared by subjecting 1000 mL of tap water to plasma treatment for 30 min (Figure 8b). Plasma was generated as a jet using nitrogen as a feed gas (99.9% N_2_, gas flow 10 L/min) with CTP-2000K low-temperature plasma device (Suman Electronics Co., Ltd., Nanjing, China), composed of a voltage regulator, a power supply (220 V/50 Hz, 40 kV, 0.33 A), and a plasma torch. The plasma jet was positioned above the surface of water at a distance of 2–3 mm (Figure 9a). The temperature of the plasma plume was estimated to be 20.7 °C, and the temperature of the water did not exceed 20 °C after 30 min of treatment. The chemical composition and characteristics of the thus-generated plasma were confirmed using a fibre-coupled spectrometer for OES (NewSpec Pty Ltd., Myrtle bank, Australia), with a resolution of 0.7 nm in a wavelength range of 200–900 nm. Optical emission spectra (OES) (Figure 9b)were collected by placing an optical fibre in close proximity to the liquid surface and 3–5 mm away from the plasma jet. PAW was applied to seeds/plants immediately after plasma treatment to take maximum advantage of the plasma-generated species, such as hydroxyl radicals and ozone, as storage is known to lead to a loss of biochemical activity in PAW.

### 3.2. Physico-Chemical Characterisation of Water

A WQ100 digital water monitoring system (BudMore Pty Ltd., Canberra, Australia) was used to quantify the dissolved oxygen, total nitrogen/ammonia ion, conductivity, pH, and temperature of the three water types. WQ100 is a commercial system designed specifically for use in agriculture and aquaculture. Its pH sensor has a measuring range of 0 to 14 pH, a resolution of 0.01 pH, a precision of ±0.1 pH, and temperature compensation with an accuracy of ±0.3 °C. The dissolved oxygen (DO) sensor has a measuring range of 0 to 20 mg/L, equivalent to 0–200% saturation at 25 °C, with a resolution of 0.01 mg/L for DO concentration and 0.1 °C for temperature. The precision of this sensor was ±2% of full scale (F.S.) for DO and ±0.3 °C for temperature. For measuring water salinity or total ionic content, an electrical conductivity sensor was employed. It had a measuring range of 0 to 200.00 µS/cm, with a resolution of 0.1 µS/cm. The precision of this sensor was ±1.5% of full scale, with a temperature precision of ±0.3 °C. Lastly, the ammonium ion (NH_4_^+^) sensor had a measuring range of 0 to 1000 mg/L, with a resolution of 0.1 mg/L and a precision of ±10% or ±1 mg/L, whichever was greater, and included a temperature precision of ±0.5 °C.

A Thermo Fisher iCap RQ quadrupole inductively coupled plasma mass spectrometer (ICP-MS) was used to investigate the elemental composition of dissolved minerals in NW, PAW, and APW. In order to stabilise the analyte elements in the liquid sample, samples were acidified by adding concentrated nitric acid to achieve a concentration of 2% *v*/*v* HNO_3_. A 10-fold dilution was then performed to reduce the influence of matrix components on the ionisation process in the plasma by reducing their concentrations. For the 10-fold dilution, 0.5 mL of the acidified sample was added to 4.95 mL of 2% HNO_3_. The results were corrected to account for these dilution factors.

To determine the concentration of hydrogen peroxide in PAW, Titanium (IV) oxysulphate reagent was first prepared by dissolving 0.75 mL of titanium (IV) oxysulphate solution in 25 mL of sulfuric acid, followed by dilution to a final volume of 100 mL with deionised water. A stock solution of H_2_O_2_ was prepared by diluting 20 µL of concentrated (~30%, ~9.8 M) H_2_O_2_ to 100 mL with deionised water and then used to prepare the working standards. The absorbance of the standards and PAW samples was measured at 410 nm using a UV–Vis spectrophotometer. A calibration curve was constructed by plotting absorbance against standard concentrations. For each measurement, 5 mL of PAW was mixed with 10 mL of titanium oxysulphate reagent, forming a yellow complex. The hydrogen peroxide concentration was calculated from the calibration curve as absorbance = m[H_2_O_2_] + c.

The dissolved ozone concentration was determined using the Beer–Lamber law, where absorbance = ε × c × l, where absorbance is UV absorption at 258 nm (measured using a UV-2450 UV/VIS spectrophotometer, Shimadzu, Japan), l is the path length of a cuvette of 1 cm, and the molar absorptivity coefficient (ε) is 2900 M^−1^ cm^−1^, as described in references [101,102,103].

### 3.3. Seed Soaking and Planting

Seeds of *B. oleracea* and *M. sativa* were purchased from Mr. Fothergill’s seeds, Australia and used without further modification.

Prior to sowing, all seeds were subjected to soaking by placing a minimum of 120 seeds into a petri dish and adding 6 mL of NW, PAW, or APW (Figure 7c and Figure S1). The seeds were allowed to soak for 3 h, uncovered, under ambient conditions.

After soaking, the seeds were immediately transferred into 16-compartment plastic growth trays, with each compartment measuring 3.5 × 4 × 5 cm and filled with ~60–65 cm^3^ of soil. In every experiment, a total of nine trays were used, corresponding to nine regimens of soaking + irrigation, namely NW soaking + NW irrigation (control), NW soaking + APW irrigation, NW soaking + PAW irrigation, APW soaking + NW irrigation, APW soaking + APW irrigation, APW soaking + PAW irrigation, PAW soaking + NW irrigation, PAW soaking + APW irrigation, and PAW soaking + PAW irrigation. The soaked seeds were sown at a depth of 3 mm, with five seeds per compartment, thus providing 120 each of *B. oleracea* and *M. sativa* seeds for each of the nine soaking + irrigation regimens. Each growth tray was placed in its own irrigation tray, which was filled with NW, APW, or PAW on the day of sowing and then topped up regularly to meet the demand of the growing plants. The plants were grown for 40 Martian days (sols) under controlled conditions, under a relative light intensity of 590 µmol/m^2^/s, the sol duration set to ~24 h 40 min, and a temperature regimen of 20 °C (12 h 20 min)/12 °C (12 h 20 min), with a gradual transition between the max and min temperatures.

On sol 10 of the experiment, 2 mL of Manutec Nitroplus hydroponic nutrient (Manutec Pty Ltd., Cavan, Australia) was added to each kit, which was 20% of the recommended amount. This nutrient contains 8% N, 1.6% P, 3% K, 3.4% Ca, and 0.5% Mg, and some trace elements.

### 3.4. Characterisation of Plant Development

On sol 40 of the experiment, the plants were removed from their growth containers and separated into stems, leaves, and roots to assess architectural differences. Shoots were weighed fresh on an ADAM Nimbus analytical balance (Adam Equipment (S.E. ASIA) Pty. Ltd., Perth, Australia) to determine the edible biomass fractions in each set. Each fraction was then oven-dried at 70 °C for 48 h, after which the dry weights were recorded to quantify the moisture-free edible and inedible biomass. The roots were fully tangled in soil, making the separation and calculation harder.

Leaf area was measured using Leaf Analyzer (https://github.com/squashking/Leaf-Analyzer, accessed on 15 July 2025), a computer-vision tool from the Australian Plant Phenomics Network that automatically extracts traits such as leaf count, dimensions, perimeter, and area. Individual leaves were arranged on a white reference board printed with the Analyser’s calibration pattern, then flattened under a transparent lid to improve accuracy. RGB (red-blue-green) photographs were taken with an iPhone 14 Pro, uploaded to the software, and processed to quantify the number of primary and secondary leaves, their total and individual areas (including the largest leaf in each category), and percentage damage from insects or disease.

The chlorophyll content was estimated using a CCM-300 Chlorophyll Content Meter (Opti-sciences, Inc., Hudson, NH, USA), which measures the fluorescence emission ratio of intensity at 735 nm/700 nm (chlorophyll fluorescence ratio) with a resolution of 1 mg/m^2^ (0.01 CFR), where 460 nm blue LED with a half bandwidth of 15 nm is used as a light source. The non-destructive technique is well suited for small measurement areas (*d* < 3 mm), such as was the case in this study.

On sol 10 of the growth experiment, stems were randomly selected for xylem vessel analysis. Using a sharp blade, cross-sections were cut between the first and second leaf nodes to preserve tissue integrity and immediately mounted on glass slides to prevent dehydration. Images were captured on a Zeiss Axio Observer microscope (Carl Zeiss Microscopy Deutschland GmbH, Oberkochen, Germany) under brightfield illumination at 5× and 10× magnification.

### 3.5. Statistical Analysis

Where multiple measurements were taken, the results were expressed in terms of their mean values and the corresponding standard deviations (SDs). Statistical data processing was performed using Student’s two-tailed *t*-tests, with a significance level of 0.05.

## 4. Conclusions

In an effort to improve the success of crops grown in soils based on simulated Martian regolith, this study examined the effects of nine soaking and irrigation regimens using various combinations of normal water (NW), plasma-activated water (PAW) enriched with biochemically active RONS, and water enriched with minerals from feldspar along with the presence of nano- and micro-bubbles (APW) on the growth performance, physiological response, and anatomical development of *M. sativa* and *B. oleracea*. Our results suggest that the magnitude of observable change in response to the choice of soaking and irrigation regimen delivered varied significantly between the species, as did the affected crop parameters, suggesting the importance of timing the delivery of RONS stimulation and mineral supplementation to meet differing plant needs at different stages of their developmental lifecycle. Within the parameters chosen in this study, for *B. oleracea*, PAW soaking and PAW irrigation provided the best overall outcome by promoting early germination and shoot growth due to breakdown of the hard seed coat by RONS, delivering mild oxidative priming which stimulated early growth, with subsequent enhanced nutrient uptake and oxidative stress tolerance from PAW at the later stages of growth. Exposure to APW during soaking and irrigation was also found to strongly promote biomass accumulation. In contrast, *M. sativa* responded more positively to PAW soaking and NW irrigation, indicating species-specific responses possibly due to seed coat permeability and intrinsic stress tolerance. PAW irrigation was also found to maintain or enhance the accumulation of photosynthetic pigments, which was probably the result of improved nitrogen assimilation and cellular redox balance. Plants exposed to PAW soaking and irrigation, particularly *M. sativa*, exhibited better-developed vascular bundles, suggesting enhanced internal water and nutrient transport mechanisms underlying the observed growth improvements.

Our experiments revealed that, while both PAW and APW can have significant positive or neutral effects on a variety of growth indicators, excessive RONS and/or mineral supplementation, particularly at specific stages of the plant lifecycle, may have negative effects in the form of hindered plant development, and thus reduced crop performance. In our study, the use of PAW for seed soaking had the tendency to enhance germination percentages and uniformity, likely due to mild oxidative stimulation by RONS that activates seed metabolism and break dormancy. The use of mineral supplementation, i.e., potassium, calcium, and soluble silica, via APW irrigation at the later stages of plant development promoted leaf expansion, chlorophyll preservation, and overall biomass accumulation, with nano-bubbles promoting nutrient solubilisation and uptake. In contrast, under certain soaking and irrigation regimens, persistent and/or excessive exposure to RONS in PAW, particularly hydroxyl radicals, ozone, and hydrogen peroxide, was more likely to saturate the plant’s antioxidant defence system, leading to oxidative stress, peroxidation of membrane lipids, and inhibition of root meristem activity. Similarly, prolonged or concentrated exposure to minerals in APW, enriched with potassium, calcium, and soluble silica from feldspar, can elevate electrical conductivity and alkalinity to levels that are disruptive to nutrient balance by causing ion toxicity or osmotic stress. These adverse effects were more pronounced when treatments were applied at sensitive developmental stages, pointing to dosage, frequency, and timing as key factors. These findings emphasise the need for stage-optimum treatment regimens that maximise benefits without inducing phytotoxicity.

Furthermore, prolonged exposure to stress factors may compromise the quality of seeds collected from these crops, e.g., via accumulation of DNA damage, thus potentially compromising the premise of self-sustaining agricultural systems at remote outposts. Future optimisation efforts should focus on improving the understanding of the individual, synergistic, and antagonistic effects delivered by specific reactive oxygen and nitrogen species and feldspar-derived minerals on plants, the effect of their absolute and relative concentrations on plant growth, and the interactions among PAW, APW, and regolith. Future works will also explore the integration of plant-beneficial microbial inoculants, such as nitrogen-fixing *Rhizobium* spp., phosphorus-solubilising *Bacillus* spp., and growth-promoting fungi like *Trichoderma* spp., with PAW and APW treatments to evaluate the potential synergistic effects on nutrient availability, root development, and overall plant resilience.

## Figures and Tables

**Table 1:** Water quality parameters and elemental composition of NW, PAW, and APW. ICP-MS data is expressed as parts per billion. BDL stands for below detection limit.

Water Quality Parameter	Water Type		
	NW	PAW	APW
pH	6.9	6.0	7.7
DO (mg/L)	2.3	0.3	2.4
Conductivity (mS/cm)	BDL	BDL	55
Elemental composition (ppb)			
^24^Mg	2751.9	1055.2	3589.7
^39^K	0.8	18.7	49.8
^44^Ca	0.4	5.3	12.5
^27^Al	28.0	26.6	33.5
^31^P	725.4	864.7	1036.7
^51^V	0.01	0.01	0.01
^55^Mn	6.0	2.4	3.3
^57^Fe	6.5	2.6	5.8
^59^Co	0.3	0.05	0.07
^60^Ni	1.2	0.9	0.6
^65^Cu	35.2	36.7	60.1
^66^Zn	11.7	9.7	7.4
^111^Cd	0.02	0.02	BDL

**Table 2:** Possible mechanisms responsible for the changes in biomass accumulation and water content in *M. sativa* and *B. oleracea* in response to the different soaking and irrigation regimens. Values are presented as mean ± SD.

Soaking	Irrigation	Avg. Biomass Weight ± SD (mg)	% Change with Regard to Control	Avg. Water Content ± SD (mg)	% Change with Regard to Control	Drivers of Change
***M. sativa***						
NW	NW	1.96 ± 0.2		13.16 ± 2.6		Control.
PAW	NW	2.49 ± 0.4	+26.8%	15.13 ± 3.2	+14.9%	ROS/RNS priming enhances growth-promoting signalling and aquaporin function.
APW	NW	2.05 ± 0.2	+4.7%	13.16 ± 2.9	−0.05%	Mineral supplementation during soaking weakly promotes nutrient uptake.
NW	PAW	1.89 ± 0.4	−3.5%	11.82 ± 3.0	−10.2%	Oxidative stress during irrigation is responsible for stress-induced membrane impairment.
PAW	PAW	1.97 ± 0.3	+0.7%	9.48 ± 2.7	−28%	Sustained ROS/RNS exposure reduces water retention due to oxidative stress.
APW	PAW	2.4 ± 0.2	+22.7%	13.84 ± 2.9	+5.1%	Improved nutrient/water uptake due to synergy between early mineral supplementation and RONS stress.
NW	APW	2.27 ± 0.2	+13.4%	12.34 ± 2.6	−6.3%	Mineral supplementation during irrigation promotes biomass accumulation but leads to mild osmotic stress.
PAW	APW	2.1 ± 0.1	+7.0%	14.25 ± 2.3	+8.2%	Synergy from early ROS/RNS priming and sustained nutrient supplementation.
APW	APW	3.3 ± 0.7	+66.9%	17.56 ± 2.9	+33.3%	Sustained mineral supplementation with micro-bubbles provides nutrient-driven growth and optimal osmotic balance by improving nutrient availability and root oxygenation.
***B. oleracea***						
NW	NW	0.54 ± 0.17		7.56 ± 2.9		Control.
PAW	NW	1.18 ± 0.15	+117.8%	14.03 ± 3.0	+85.2%	Moderate ROS/RNS priming triggers antioxidant defence and aquaporin expression.
APW	NW	1.78 ± 0.2	+216.7%	22.39 ± 3.2	+196.1%	Increased K^+^ and Ca^2+^ promote cell wall stability, osmotic adjustment, and chlorophyll formation.
NW	PAW	3.14 ± 0.4	+477%	33.52 ± 2.8	+343.3%	Sustained RONS-mediated growth-promoting signalling and stomatal opening.
PAW	PAW	2.99 ± 0.2	+450.6%	29.14 ± 3.18	+285.4%	Oxidative priming and ROS/RNS-mediated stimulation of physiological pathways.
APW	PAW	1.98 ± 0.2	+264.3%	21.2 ± 3.3	+180.6%	Early mineral priming and sustained RONS stimulation balance nutrient delivery and stress signalling.
NW	APW	2.15 ± 0.2	+294.7%	25.6 ± 2.9	+238.2%	Sustained delivery of K^+^, Ca^2+^, and silicates improve osmotic balance, cell wall strength, and photosynthesis.
PAW	APW	2.54 ± 0.2	+373%	30.45 ± 3.5	+302.6%	Synergy from oxidative seed priming and ROS signalling and sustained delivery of mineral nutrients with the help of micro-bubbles.
APW	APW	1.1 ± 0.1	+96%	12.9 ± 2.9	+70.1%	Early mineral priming and sustained delivery of mineral nutrients through micro-bubbles support enhanced grow and water retention.

**Table 3:** Possible mechanisms responsible for changes in the seed germination of *M. sativa* and *B. oleracea* in response to the different soaking and irrigation regimens. Values are presented as mean ± SD.

Soaking	Irrigation	Germination (%)	%Change with Regard to Control	Drivers of Change
***M. sativa***				
NW	NW	30.0		Control.
PAW	NW	20.0	−33%	Excess oxidative stress from ROS during soaking.
APW	NW	7.5	−75%	Osmotic or ionic stress from leached mineral content. Higher ionic load, disrupting the water imbibition and enzymatic activation required in germination.
NW	PAW	30.0	0%	ROS-mediated signalling during irrigation increased membrane permeability and improved water retention during the early stages of development.
PAW	PAW	42.5	+42%	ROS exposure during soaking and irrigation induced antioxidant enzyme systems, enhanced early energy metabolism, and improved stress tolerance.
APW	PAW	35.0	+17%	Soaking in APW augmented ion availability. PAW irrigation promoted metabolic activation and physiological revival.
NW	APW	32.5	+8%	The post-sowing delivery of minerals such as potassium and calcium may have supported root activation and nutrient transport and enhanced seedling establishment.
PAW	APW	27.5	−8%	Oxidative priming during soaking. The minerals in APW may disrupt redox homeostasis or cause osmotic stress, disrupting seedling recovery.
APW	APW	37.5	+25%	Sustained delivery of mineral supplementation at ionic concentrations within a favourable physiological range and the presence of micro-bubbles.
***B. oleracea***				
NW	NW	7.5		Control.
PAW	NW	7.5	0%	Oxidative priming through RONS exposure during soaking. Lack of sustained supply of RONS during irrigation. A one-stage oxidative treatment is not sufficient.
APW	NW	12.5	+67%	Increased availability of major ions such as Ca, Mg, and K during early hydration, which enabled nutrient activation mechanisms during seed imbibition.
NW	PAW	27.5	+267%	Sustained delivery of RONS enhanced stress tolerance, permeability of cells, and enzyme activation during early seedling establishment.
PAW	PAW	30.0	+300%	Consistent oxidative stimulation significantly enhanced metabolic readiness and promoted root initiation and shoot emergence in plants under abiotic stress.
APW	PAW	17.5	+133%	Increased nutrient availability during soaking. RONS during irrigation supported stress adaptation and metabolic recovery. Presence of micro-bubbles.
NW	APW	2.5	−67%	Osmotic or ionic stress from minerals in APW, interfering with water entry and cell division during germination.
PAW	APW	15.0	+100%	Oxidative priming during soaking. Sustained supply of mineral nutrients during seedling establishment.
APW	APW	7.5	0%	In the absence of RONS priming that facilitates water uptake by seeds, sustained mineral supplementation via APW irrigation has limited impact.

**Table 4:** Possible mechanisms responsible for changes in the shoot length of *M. sativa* and *B. oleracea* in response to the different soaking and irrigation regimens. Values are presented as mean ± SD.

Soaking	Irrigation	Shoot Length ± SD (cm)	% Change with Regards to Control	Drivers of Change
***M. sativa***				
NW	NW	17.3 ± 5.1		Control.
PAW	NW	16.2 ± 7.6	−6.3%	Lack of post-sowing supplementation through irrigation likely limited shoot elongation. Early benefits from PAW priming were not sustained.
APW	NW	15.7 ± 5	−9.4%	Stimulation from e.g., Ca or K during soaking was potentially offset by osmotic imbalances or toxic ion accumulation restricting shoot expansion.
NW	PAW	17.1 ± 5.7	−1.6%	PAW irrigation enhanced post-sowing water availability and triggered beneficial oxidative signalling pathways, aiding elongation even without priming.
PAW	PAW	15.6 ± 5.2	−9.6%	Combined oxidative and osmotic stress.
APW	PAW	17.9 ± 4.4	+3.8%	PAW irrigation may have counteracted the ionic stress of APW soaking, facilitating ROS-mediated signalling and improved nutrient uptake.
NW	APW	15.9 ± 5.5	−7.8%	Post-germination mineral exposure supported secondary growth processes, e.g., cell wall reinforcement. Lack of early priming.
PAW	APW	15.2 ± 5.5	−11.8%	PAW soaking might have enhanced germination metabolism, but APW irrigation likely introduced ionic imbalance or stress, reducing overall shoot elongation.
APW	APW	17.2 ± 6.2	−0.8%	Lack of oxidative priming. Sustained mineral supplementation supported only moderate shoot development.
***B. oleracea***				
NW	NW	2.8 ± 1.6		Control.
PAW	NW	2.6 ± 1.4	−6%	Oxidative priming alone was ineffective without sustained RONS or mineral supplementation during irrigation to offset a nutrient-poor environment.
APW	NW	3.0 ± 1.7	+9%	Mineral supplementation during soaking supported early metabolic activity, and lack of sustained supplementation during irrigation reduced overall benefit.
NW	PAW	3.9 ± 1.5	+41%	PAW irrigation post-sowing promoted enhanced water uptake and antioxidant activity, improving shoot development despite the lack of priming.
PAW	PAW	4.0 ± 2.6	+44%	Oxidative priming from PAW soak and continued exposure through irrigation likely activated defence enzymes and improved nutrient mobilisation.
APW	PAW	3.6 ± 1.6	+30%	PAW irrigation helped buffer any ionic imbalance introduced by APW soaking, aiding recovery and growth.
NW	APW	3.8 ± 1.9	+37%	Mineral-rich APW irrigation likely provided key nutrients like K^+^, Ca^2+^, and P during active growth phases, improving shoot length even without priming.
PAW	APW	3.0 ± 2.1	+9%	Although oxidative priming was applied, APW irrigation may have introduced ionic stress or lacked a synergistic ROS response, limiting effectiveness.
APW	APW	3.4 ± 1.3	+23%	Moderate success may reflect cumulative mineral supply, aiding later-stage growth, but without priming, metabolic activation may have lagged.

**Table 5:** Possible mechanisms responsible for changes in the leaf area of *M. sativa* and *B. oleracea* in response to the different soaking and irrigation regimens. Values are presented as mean ± SD.

Soaking	Irrigation	Leaf Area ± SD (mm^2^)	% Change with Regard to Control	Drivers of Change
***M. sativa***				
NW	NW	791 ± 147		Control.
PAW	NW	494 ± 79	−37%	Oxidative stress during soaking.
APW	NW	627 ± 110	−20%	Excess mineral supplementation during soaking.
NW	PAW	592 ± 72	−25%	Sustained oxidative stress during irrigation.
PAW	PAW	633 ± 89	−20%	Sustained oxidative stress during seed soaking and irrigation.
APW	PAW	705 ± 86	−11%	Possible antagonistic action between excess mineral supplementation during soaking and sustained oxidative stress during irrigation.
NW	APW	632 ± 57	−20%	Sustained delivery of excess minerals during irrigation.
PAW	APW	734 ± 137	−7.2%	Possible antagonistic action between oxidative stress during soaking and sustained mineral supplementation during irrigation.
APW	APW	642 ± 160	−18.8%	Sustained exposure to excess minerals during soaking and irrigation.
***B. oleracea***				
NW	NW	3368 ± 232		Control
PAW	NW	2640 ± 412	−21%	Oxidative stress during seed germination.
APW	NW	2420 ± 129	−28%	Excess minerals affected embryonic leaf tissue by altering water potential and ion balance, restricting turgor-supported leaf cell growth.
NW	PAW	2906 ± 326	−13%	Sustained exposure to RONS post-germination enhances nutrient uptake and stimulates cell signalling for growth.
PAW	PAW	3440 ± 392	+2%	RONS only weakly stimulate cell division, enzyme activation, and uptake of nutrients, as well as increased uptake of water and redox signalling.
APW	PAW	2832 ± 562	−27%	Sustained RONS stimulation during irrigation is insufficient to fully offset the ionic stress introduced during APW soaking.
NW	APW	2450 ± 380	−27%	Elevated ion concentrations in APW lead to osmotic or ionic stress, hindering water balance and cell enlargement in leaf tissues.
PAW	APW	2939 ± 342	−13%	RONS activation of early metabolic and oxidative pathways that promoted initial seedling development is offset by mineral stress during irrigation.
APW	APW	2832 ± 565	−16%	Sustained mineral stress reduces the overall photosynthetic surface area.

**Table 6:** Possible mechanisms responsible for the changes in chlorophyll accumulation in *M. sativa* and *B. oleracea* in response to the different soaking and irrigation regimens. Values are presented as mean ± SD.

Soaking	Irrigation	Chlorophyl Concentration ± SD (mg/m^2^)	% Change with Regard to Control	Drivers of Change
***M. sativa***				
NW	NW	371 ± 27		Control.
PAW	NW	385 ± 13	+3.8%	ROS/RNS exposure during seed soaking mildly activates pathways for chlorophyll biosynthesis, with the potential to increase pigment density.
APW	NW	387 ± 37	+4.3%	Mineral priming may support chlorophyll assembly in developing chloroplasts, boosting pigment content during germination.
NW	PAW	394 ± 29	+6.2%	Sustained ROS/RNS stimulation drives continued up-regulation of chlorophyll synthesis.
PAW	PAW	408 ± 22	+10%	Sustained ROS/RNS exposure during soaking and irrigation may synergistically prolong activation of key biosynthetic enzymes.
APW	PAW	408 ± 31	+10%	Early mineral support for chloroplast development, followed by ROS/RNS-driven enhancement of chlorophyll synthesis in maturing tissues.
NW	APW	401 ± 9	+8.1%	Sustained mineral delivery during leaf expansion increases chlorophyll content by improving enzyme cofactor availability.
PAW	APW	406 ± 19	+9.4%	Oxidative seed priming preparing tissues to capitalise on later nutrient supply from mineral-rich irrigation, cumulatively enhancing chlorophyll accumulation.
APW	APW	420 ± 9	+13.2%	Mineral enrichment throughout germination and growth promotes chlorophyll biosynthesis to maximise pigment density.
***B. oleracea***				
NW	NW	315 ± 37		Control.
PAW	NW	385 ± 13	+22%	RONS in the PAW used for soaking preconditioned seed metabolism to hyper-regulate chlorophyll biosynthesis.
APW	NW	348 ± 26	+10.5%	Mineral priming during soaking induces enzyme cofactors required for chlorophyll biosynthesis. pH or ionic strength changes may temporarily inhibit chloroplast development or result in heterogeneous mineral uptake among seeds.
NW	PAW	359 ± 55	+14%	Sustained delivery of stimulating RONS into developing leaves and roots, causing long-term chlorophyll accumulation.
PAW	PAW	352 ± 69	+11.7%	Sustained RONS-mediated activation of chlorophyll-producing pathways. Excess RONS stress can lead to some pigment loss/degradation, reflected in higher SD.
APW	PAW	379 ± 24	+20.3%	Mineral priming for early development of chlorophyll. Sustained delivery of reactive species enhanced the content of chlorophyll in leaves.
NW	APW	361 ± 31	+14.6%	Sustained delivery of minerals at the leaf expansion stage increases chlorophyll through direct nutrient support.
PAW	APW	370 ± 15	+17.5%	Initial oxidative treatment sensitising plant metabolism to make use of the sustained supplementation with K^+^/Ca^2+^ with irrigation.
APW	APW	346 ± 28	+9.8%	Sustained delivery of mineral cofactors for chlorophyll biosynthesis. Long-term high ion concentration can result in precipitation of essential micronutrients (e.g., Mg^2+^) or cause imbalances that limit further pigment accumulation.

## Data Availability

All data collected during the studies are included in this paper.

## Supplementary Materials

The following supporting information can be downloaded at: https://www.mdpi.com/article/10.3390/ijms26178318/s1.

## References

[1] Eichler A., Hadland N., Pickett D., Masaitis D., Handy D., Perez A., Batcheldor D., Wheeler B., Palmer A. (2021). Challenging the agricultural viability of martian regolith simulants. Icarus.

[2] Neukart F. (2024). Towards sustainable horizons: A comprehensive blueprint for Mars colonization. Heliyon.

[3] Levchenko I., Goebel D., Pedrini D., Albertoni R., Baranov O., Kronhaus I., Bazaka K. (2025). Recent innovations to advance space electric propulsion technologies. Prog. Aerosp. Sci..

[4] Levchenko I., Baranov O., Keidar M., Riccardi C., Roman H.E., Xu S., Alexander K. (2024). Additive technologies and materials for the next-generation CubeSats and small satellites. Adv. Funct. Mater..

[5] Duri L.G., Caporale A.G., Rouphael Y., Vingiani S., Palladino M., De Pascale S., Adamo P. (2022). The potential for lunar and martian regolith simulants to sustain plant growth: A multidisciplinary overview. Front. Astron. Space Sci..

[6] Gilster P. (2023). Food production on Mars: Dirt farming as the most scalable solution for settlement. Centauri Dreams.

[7] Cheatham R.W., Sultana A.I., Reza M.T. (2025). Co-activation of Martian regolith and hydrochar for enhanced water retention and water holding capacity. J. Anal. Appl. Pyrolysis.

[8] Maity T., Saxena A. (2024). Challenges and innovations in food and water availability for a sustainable Mars colonization. Life Sci. Space Res..

[9] Wamelink G., Frissel J., Krijnen W., Verwoert M. (2019). Crop growth and viability of seeds on Mars and Moon soil simulants. Open Agric..

[10] Kyriacou M.C., De Pascale S., Kyratzis A., Rouphael Y. (2017). Microgreens as a component of space life support systems: A cornucopia of functional food. Front. Plant Sci..

[11] Fabek Uher S., Radman S., Opačić N., Dujmović M., Benko B., Lagundžija D., Mijić V., Prša L., Babac S., Šic Žlabur J. (2023). Alfalfa, cabbage, beet and fennel microgreens in floating hydroponics—Perspective nutritious food?. Plants.

[12] Rainwater R., Mukherjee A. (2021). The legume-rhizobia symbiosis can be supported on Mars soil simulants. PLoS ONE.

[13] Harris F., Dobbs J., Atkins D., Ippolito J.A., Stewart J.E. (2021). Soil fertility interactions with Sinorhizobium-legume symbiosis in a simulated Martian regolith; effects on nitrogen content and plant health. PLoS ONE.

[14] Wang X.-Q., Zhou R.-W., Groot G.d., Bazaka K., Murphy A.B., Ostrikov K. (2017). Spectral characteristics of cotton seeds treated by a dielectric barrier discharge plasma. Sci. Rep..

[15] Zhou R., Zhou R., Zhang X., Zhuang J., Yang S., Bazaka K., Ostrikov K. (2016). Effects of Atmospheric-Pressure N_2_, He, Air, and O_2_ Microplasmas on Mung Bean Seed Germination and Seedling Growth. Sci. Rep..

[16] Asghari A., Sabbaghtazeh E., Milani N.R., Kouhi M., Maralani A.A., Gharbani P., Khiaban A.S. (2025). Effects of plasma-activated water on germination and initial seedling growth of wheat. PLoS ONE.

[17] Su J., Liu Y., Han F., Gao F., Gan F., Huang K., Li Z. (2024). ROS, an Important Plant Growth Regulator in Root Growth and Development: Functional Genes and Mechanism. Biology.

[18] Domonkos M., Tichá P., Trejbal J., Demo P. (2021). Applications of cold atmospheric pressure plasma technology in medicine, agriculture and food industry. Appl. Sci..

[19] Yan D., Lin L., Zvansky M., Kohanzadeh L., Taban S., Chriqui S., Keidar M. (2022). Improving seed germination by cold atmospheric plasma. Plasma.

[20] Pańka D., Jeske M., Łukanowski A., Baturo-Cieśniewska A., Prus P., Maitah M., Maitah K., Malec K., Rymarz D., Muhire J.d.D. (2022). Can cold plasma be used for boosting plant growth and plant protection in sustainable plant production?. Agronomy.

[21] Sasi S., Prasad K., Weerasinghe J., Bazaka O., Ivanova E.P., Levchenko I., Bazaka K. (2023). Plasma for aquaponics. Trends Biotechnol..

[22] Wang X.-Q., Wang F.-P., Chen W., Huang J., Bazaka K., Ostrikov K. (2016). Non-equilibrium plasma prevention of Schistosoma japonicum transmission. Sci. Rep..

[23] Zhou R., Zhou R., Zhang X., Li J., Wang X., Chen Q., Yang S., Chen Z., Bazaka K., Ostrikov K. (2016). Synergistic Effect of Atmospheric-pressure Plasma and TiO_2_ Photocatalysis on Inactivation of *Escherichia coli* Cells in Aqueous Media. Sci. Rep..

[24] Recek N., Zhou R., Zhou R., Te’o V.S.J., Speight R.E., Mozetič M., Vesel A., Cvelbar U., Bazaka K., Ostrikov K. (2018). Improved fermentation efficiency of S. cerevisiae by changing glycolytic metabolic pathways with plasma agitation. Sci. Rep..

[25] Radoslovich E. (1975). Feldspar minerals. Soil Components: Volume 2: Inorganic Components.

[26] Jundullah Hanafi M.I., Murshed M.M., Robben L., Gesing T.M. (2025). Plagioclase feldspars (Ca_1−x_ Na_x_)(Al_2−x_ Si_2+x_) O_8_: Synthesis and characterizations of mechanical weathering relevant to Martian regolith. Z. Krist.-Cryst. Mater..

[27] What Is Aquapulse. https://www.aquapulse.technology/.

[28] Weller R. Improved Marigold Bud and Flower Growth with Innovative Irrigation. https://www.floraldaily.com/article/9716249/improved-marigold-bud-and-flower-growth-with-innovative-irrigation/.

[29] Peyrusson F. (2021). Hydrogels improve plant growth in mars analog conditions. Front. Astron. Space Sci..

[30] Rogers A.D., Nekvasil H. (2015). Feldspathic rocks on Mars: Compositional constraints from infrared spectroscopy and possible formation mechanisms. Geophys. Res. Lett..

[31] Levchenko l., Xu S., Baranov O., Bazaka K. (2024). How to Survive at Point Nemo? Fischer–Tropsch, Artificial Photosynthesis, and Plasma Catalysis for Sustainable Energy at Isolated Habitats. Glob. Chall..

[32] Dai X., Bazaka K., Richard D.J., Thompson E.W., Ostrikov K. (2018). The Emerging Role of Gas Plasma in Oncotherapy. Trends Biotechnol..

[33] Ishaq M., Bazaka K., Ostrikov K. (2015). Intracellular effects of atmospheric-pressure plasmas on melanoma cancer cells. Phys. Plasmas.

[34] Zhou R., Zhou R., Wang S., Mihiri Ekanayake U.G., Fang Z., Cullen P.J., Bazaka K., Ostrikov K. (2020). Power-to-chemicals: Low-temperature plasma for lignin depolymerisation in ethanol. Bioresour. Technol..

[35] Zhou R., Zhou R., Zhang X., Fang Z., Wang X., Speight R., Wang H., Doherty W., Cullen P.J., Ostrikov K. (2019). High-Performance Plasma-Enabled Biorefining of Microalgae to Value-Added Products. ChemSusChem.

[36] Thirumdas R., Kothakota A., Annapure U., Siliveru K., Blundell R., Gatt R., Valdramidis V.P. (2018). Plasma activated water (PAW): Chemistry, physico-chemical properties, applications in food and agriculture. Trends Food Sci. Technol..

[37] Liao X., Xiang Q., Cullen P.J., Su Y., Chen S., Ye X., Liu D., Ding T. (2020). Plasma-activated water (PAW) and slightly acidic electrolyzed water (SAEW) as beef thawing media for enhancing microbiological safety. LWT.

[38] Lamichhane P., Paneru R., Nguyen L.N., Lim J.S., Bhartiya P., Adhikari B.C., Mumtaz S., Choi E.H. (2020). Plasma-assisted nitrogen fixation in water with various metals. React. Chem. Eng..

[39] Li S., Medrano J.A., Hessel V., Gallucci F. (2018). Recent progress of plasma-assisted nitrogen fixation research: A review. Processes.

[40] Adhikari B., Adhikari M., Park G. (2020). The effects of plasma on plant growth, development, and sustainability. Appl. Sci..

[41] Sarinont T., Amano T., Attri P., Koga K., Hayashi N., Shiratani M. (2016). Effects of plasma irradiation using various feeding gases on growth of *Raphanus sativus* L.. Arch. Biochem. Biophys..

[42] Takamatsu T., Uehara K., Sasaki Y., Miyahara H., Matsumura Y., Iwasawa A., Ito N., Azuma T., Kohno M., Okino A. (2014). Investigation of reactive species using various gas plasmas. RSC Adv..

[43] Šerá B., Scholtz V., Jirešová J., Khun J., Julák J., Šerý M. (2021). Effects of Non-Thermal Plasma Treatment on Seed Germination and Early Growth of Leguminous Plants-A Review. Plants.

[44] Dahal R., Dhakal O.B., Acharya T.R., Lamichhane P., Gautam S., Chalise R., Kaushik N., Choi E.H., Kaushik N.K. (2024). Investigating plasma activated water as a sustainable treatment for improving growth and nutrient uptake in maize and pea plant. Plant Physiol. Biochem..

[45] Li D.-X., Gan L., Bronja A., Schmitz O.J. (2015). Gas chromatography coupled to atmospheric pressure ionization mass spectrometry (GC-API-MS). Anal. Chim. Acta.

[46] Zhou R., Zhou R., Prasad K., Fang Z., Speight R., Bazaka K., Ostrikov K.K. (2018). Cold atmospheric plasma activated water as a prospective disinfectant: The crucial role of peroxynitrite. Green Chem..

[47] Jiang B., Zheng J., Qiu S., Wu M., Zhang Q., Yan Z., Xue Q. (2014). Review on electrical discharge plasma technology for wastewater remediation. Chem. Eng. J..

[48] Gururani P., Bhatnagar P., Bisht B., Kumar V., Joshi N.C., Tomar M.S., Pathak B. (2021). Cold plasma technology: Advanced and sustainable approach for wastewater treatment. Environ. Sci. Pollut. Res..

[49] Zhang X., Zhou R., Bazaka K., Liu Y., Zhou R., Chen G., Chen Z., Liu Q., Yang S., Ostrikov K. (2018). Quantification of plasma produced OH radical density for water sterilization. Plasma Process. Polym..

[50] Park J.-S., Kurata K. (2009). Application of Microbubbles to Hydroponics Solution Promotes Lettuce Growth. HortTechnology.

[51] Rogers J., Bennett P., Choi W. (1998). Feldspars as a source of nutrients for microorganisms. Am. Mineral..

[52] Silva J.C., Ulsen C., Bergerman M.G., Horta D.G. (2019). Reduction of Fe_2_O_3_ content of foyaite by flotation and magnetic separation for ceramics production. J. Mater. Res. Technol..

[53] Blum A.E., Stillings L.L., Arthur F.W., Susan L.B. (1995). Chapter 7. Feldspar Dissolution Kinetics. Chemical Weathering Rates of Silicate Minerals.

[54] Sardans J., Peñuelas J. (2021). Potassium control of plant functions: Ecological and agricultural implications. Plants.

[55] Johnson R., Vishwakarma K., Hossen M.S., Kumar V., Shackira A., Puthur J.T., Abdi G., Sarraf M., Hasanuzzaman M. (2022). Potassium in plants: Growth regulation, signaling, and environmental stress tolerance. Plant Physiol. Biochem..

[56] Poovaiah B., Reddy A., Feldman L. (1993). Calcium and signal transduction in plants. Crit. Rev. Plant Sci..

[57] Imtiaz M., Rizwan M.S., Mushtaq M.A., Ashraf M., Shahzad S.M., Yousaf B., Saeed D.A., Rizwan M., Nawaz M.A., Mehmood S. (2016). Silicon occurrence, uptake, transport and mechanisms of heavy metals, minerals and salinity enhanced tolerance in plants with future prospects: A review. J. Environ. Manag..

[58] Hoeben W.F.L.M., van Ooij P.P., Schram D.C., Huiskamp T., Pemen A.J.M., Lukeš P. (2019). On the Possibilities of Straightforward Characterization of Plasma Activated Water. Plasma Chem. Plasma Process..

[59] Galieni A., Falcinelli B., Stagnari F., Datti A., Benincasa P. (2020). Sprouts and microgreens: Trends, opportunities, and horizons for novel research. Agronomy.

[60] García-Tenesaca M.M., Llugany M., Boada R., Sánchez-Martín M.-J., Valiente M. (2024). Phytochemical Profile, Bioactive Properties, and Se Speciation of Se-Biofortified Red Radish (*Raphanus sativus*), Green Pea (*Pisum sativum*), and Alfalfa (*Medicago sativa*) Microgreens. J. Agric. Food Chem..

[61] Grela E., Pietrzak K. (2014). Production technology, chemical composition and use of alfalfa protein-xanthophyll concentrate as dietary supplement. J. Food Process. Technol..

[62] Drozdowska M., Leszczyńska T., Koronowicz A., Piasna-Słupecka E., Domagała D., Kusznierewicz B. (2020). Young shoots of red cabbage are a better source of selected nutrients and glucosinolates in comparison to the vegetable at full maturity. Eur. Food Res. Technol..

[63] Sharma S., Priyanka, Shree B., Ramachandran P., Kumar V., Thakur R., Kumar S. (2023). Cabbage and Red Cabbage Sprouts: Powerhouse of Nutrients. Advances in Plant Sprouts: Phytochemistry and Biofunctionalities.

[64] Edde P.A., Edde P.A. (2022). 11-Arthropod pests of alfalfa (*Medicago sativa* L.). Field Crop Arthropod Pests of Economic Importance.

[65] Naylor R.E.L., Thomas B. (2003). Seed development|Seed Production. Encyclopedia of Applied Plant Sciences.

[66] Diatta A.A., Min D., Jagadish S.V.K., Sparks D.L. (2021). Chapter Two-Drought stress responses in non-transgenic and transgenic alfalfa—Current status and future research directions. Advances in Agronomy.

[67] Ghareaghajlou N., Hallaj-Nezhadi S., Ghasempour Z. (2021). Red cabbage anthocyanins: Stability, extraction, biological activities and applications in food systems. Food Chem..

[68] Sarkar D., Rakshit A. (2017). Red cabbage as potential functional food in the present perspective. Int. J. Bioresour. Sci..

[69] Bian Q., Dong Z., Zhao Y., Feng Y., Fu Y., Wang Z., Zhu J., Ma L. (2025). Micro-/nanobubble oxygenation irrigation enhances soil phosphorus availability and yield by altering soil bacterial community abundance and core microbial populations. Front. Plant Sci..

[70] Appiah E.A., Balla-Kovács A., Ocwa A., Csajbók J., Kutasy E. (2024). Enhancing Alfalfa (*Medicago sativa* L.) Productivity: Exploring the Significance of Potassium Nutrition. Agronomy.

[71] Berg W.K., Brouder S.M., Cunningham S.M., Volenec J.J. (2021). Potassium and phosphorus fertilizer impacts on alfalfa taproot carbon and nitrogen reserve accumulation and use during fall acclimation and initial growth in spring. Front. Plant Sci..

[72] Singh R., Parihar P., Singh S., Mishra R.K., Singh V.P., Prasad S.M. (2017). Reactive oxygen species signaling and stomatal movement: Current updates and future perspectives. Redox Biol..

[73] Locatelli S., Triolone S., De Bonis M., Zanin G., Nicoletto C. (2025). Non-Thermal Plasma-Activated Water Enhances Nursery Production of Vegetables: A Species-Specific Study. Agronomy.

[74] Beak H.K., Priatama R.A., Han S.-I., Song I., Park S.J., Lee Y.K. (2024). Biomass enhancement and activation of transcriptional regulation in sorghum seedling by plasma-activated water. Front. Plant Sci..

[75] Guragain R.P., Pradhan S.P., Baniya H.B., Pandey B.P., Basnet N., Sedhai B., Dhungana S., Chhetri G.K., Joshi U.M., Subedi D.P. (2021). Impact of Plasma-Activated Water (PAW) on Seed Germination of Soybean. J. Chem..

[76] Ahmed S., Hayashi N. (2024). Enhancement of antioxidative potential of mung bean by oxygen plasma irradiation of seeds. Sci. Rep..

[77] Adhikari B., Adhikari M., Ghimire B., Park G., Choi E.H. (2019). Cold Atmospheric Plasma-Activated Water Irrigation Induces Defense Hormone and Gene expression in Tomato seedlings. Sci. Rep..

[78] Zhang S., Rousseau A., Dufour T. (2017). Promoting lentil germination and stem growth by plasma activated tap water, demineralized water and liquid fertilizer. RSC Adv..

[79] Sfaxi-Bousbih A., Chaoui A., El Ferjani E. (2010). Unsuitable Availability of Nutrients in Germinating Bean Embryos Exposed to Copper Excess. Biol. Trace Elem. Res..

[80] Zubair M., Shafqat A., Jabben N., Shafiq M., Balal R.M., Tahir M.A., Hashmi M.M., Naqvi S.A.A., Ali N., Abbas S.M. (2024). Enhancing cabbage resilience against heavy metal stress through silicon amendments and melatonin: A depth investigation. Sci. Hortic..

[81] Oshita S., Boerzhijin S., Kameya H., Yoshimura M., Sotome I. (2023). Promotion Effects of Ultrafine Bubbles/Nanobubbles on Seed Germination. Nanomaterials.

[82] Li L., Zhang L., Dong Y. (2025). Seed priming with cold plasma mitigated the negative influence of drought stress on growth and yield of rapeseed (*Brassica napus* L.). Ind. Crops Prod..

[83] Wang Y., Wang S., Sun J., Dai H., Zhang B., Xiang W., Hu Z., Li P., Yang J., Zhang W. (2021). Nanobubbles promote nutrient utilization and plant growth in rice by upregulating nutrient uptake genes and stimulating growth hormone production. Sci. Total Environ..

[84] Hasanuzzaman M., Bhuyan M.B., Nahar K., Hossain M.S., Mahmud J.A., Hossen M.S., Masud A.A.C., Moumita, Fujita M. (2018). Potassium: A vital regulator of plant responses and tolerance to abiotic stresses. Agronomy.

[85] Yeremko L., Czopek K., Staniak M., Marenych M., Hanhur V. (2025). Role of Environmental Factors in Legume-Rhizobium Symbiosis: A Review. Biomolecules.

[86] Alves Junior C., de Oliveira Vitoriano J., da Silva D.L.S., de Lima Farias M., de Lima Dantas N.B. (2016). Water uptake mechanism and germination of Erythrina velutina seeds treated with atmospheric plasma. Sci. Rep..

[87] Lotfy K., Al-Harbi N.A., Abd El-Raheem H. (2019). Cold atmospheric pressure nitrogen plasma jet for enhancement germination of wheat seeds. Plasma Chem. Plasma Process..

[88] Holc M., Gselman P., Primc G., Vesel A., Mozetič M., Recek N. (2022). Wettability and Water Uptake Improvement in Plasma-Treated Alfalfa Seeds. Agriculture.

[89] Zambon Y., Contaldo N., Laurita R., Várallyay E., Canel A., Gherardi M., Colombo V., Bertaccini A. (2020). Plasma activated water triggers plant defence responses. Sci. Rep..

[90] Stoleru V., Burlica R., Mihalache G., Dirlau D., Padureanu S., Teliban G.-C., Astanei D., Cojocaru A., Beniuga O., Patras A. (2020). Plant growth promotion effect of plasma activated water on Lactuca sativa L. cultivated in two different volumes of substrate. Sci. Rep..

[91] Zhao D., Oosterhuis D.M., Bednarz C.W. (2001). Influence of Potassium Deficiency on Photosynthesis, Chlorophyll Content, and Chloroplast Ultrastructure of Cotton Plants. Photosynthetica.

[92] Talebi S., Majd A., Mirzai M., Jafari S., Abedini M. (2015). The study of potassium silicate effects on qualitative and quantitative performance of potato (*Solanum tuberosum* L.). Biol. Forum.

[93] Baram S., Weinstein M., Evans J.F., Berezkin A., Sade Y., Ben-Hur M., Bernstein N., Mamane H. (2022). Drip irrigation with nanobubble oxygenated treated wastewater improves soil aeration. Sci. Hortic..

[94] Saberi M., Sanavy M., Zare R., Ghomi H. (2019). Improvement of photosynthesis and photosynthetic productivity of winter wheat by cold plasma treatment under haze condition. J. Agric. Sci. Technol..

[95] Kabir A.H., Rahman M.M., Das U., Sarkar U., Roy N.C., Reza M.A., Talukder M.R., Uddin M.A. (2019). Reduction of cadmium toxicity in wheat through plasma technology. PLoS ONE.

[96] Bailly C., Kranner I. (2011). Analyses of reactive oxygen species and antioxidants in relation to seed longevity and germination. Methods Mol. Biol..

[97] Qureshi W.A., Gao J., Elsherbiny O., Mosha A.H., Tunio M.H., Qureshi J.A. (2025). Boosting Aeroponic System Development with Plasma and High-Efficiency Tools: AI and IoT—A Review. Agronomy.

[98] Wang J., Cheng J.-H., Sun D.-W. (2023). Enhancement of wheat seed germination, seedling growth and nutritional properties of wheat plantlet juice by plasma activated water. J. Plant Growth Regul..

[99] Seleiman M.F., Ali N., Nungula E.Z., Gitari H.I., Alhammad B.A., Battaglia M.L. (2024). Enhancing germination and seedling growth of barley using plasma-activated water (PAW) with neutralized pH. Cogent Food Agric..

[100] Gibson-Corley K.N., Olivier A.K., Meyerholz D.K. (2013). Principles for valid histopathologic scoring in research. Vet. Pathol..

[101] Hart E.J., Sehested K., Holoman J. (1983). Molar absorptivities of ultraviolet and visible bands of ozone in aqueous solutions. Anal. Chem..

[102] Li J., Li X., Li K., Tao T. (2018). Plasmas ozone inactivation of Legionella in deionized water and wastewater. Environ. Sci. Pollut. Res..

[103] Eisenberg G. (1943). Colorimetric determination of hydrogen peroxide. Ind. Eng. Chem. Anal. Ed..

