# Supplementary material for: Enhanced Plant Growth on Simulated Martian Regolith via Water Chemistry Optimisation: The Role of RONS and Nano/Micro-Bubbles
Source: Int J Mol Sci. 2025 Aug 27;26(17):8318. doi: 10.3390/ijms26178318 (PMC12428210; doi:10.3390/ijms26178318)
Supplement: Supplementary file 1 [file ijms-26-08318-s001.zip › ijms-3813831-supplementary.pdf]

Article

Enhanced Plant Growth on Simulated Martian Regolith via Water Chemistry Optimisation: The Role of RONS and Nano/Micro-Bubbles

Syamlal Sasi[1], Priyanka Prakash[1], Steve Hayden[2], David Dooley[2], Richard Poiré[3], Tao Hu[3], Janith Weerasinghe[1], Igor Levchenko[4], Karthika Prasad[1,*], Katia Alexander[1,*]

[1] School of Engineering, ANU College of Systems and Society, The Australian National University, Canberra, ACT 2600, Australia. syamlal.sasi@anu.edu.au, priyanka.priyanka1@anu.edu.au, janith.adikarammudiyanselage@anu.edu.au, karthika.prasad@anu.edu.au, katia.alexander@anu.edu.au

[2] Aquapulse-Product development, Clear World Water Technology Limited, Stanmore, Middlesex HA7 4PX, United Kingdom. steve.hayden@aquapulse.tech, david.dooley@aquapulse.tech

[3] Australian Plant Phenomics Network, Australian National University, Canberra, ACT 2600, Australia. richard.poire@anu.edu.au, tao.hu@anu.edu.au

[4] Dipartimento di Fisica, Università di Milano-Bicocca, Piazza della Scienza 3, 20126 Milano, Italy. levchenko.igor@nie.edu.sg

* Correspondence: karthika.prasad@anu.edu.au (K.P.), katia.alexander@anu.edu.au (K.A.)

Supplementary Information

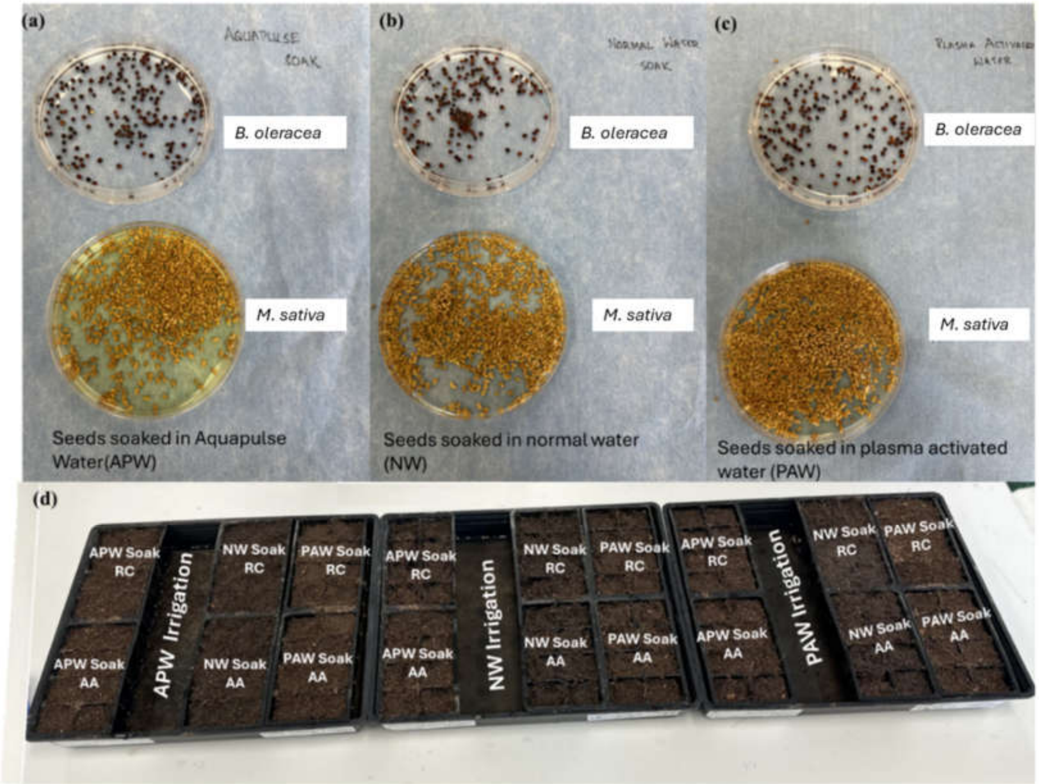

Figure S1. Images of seeds of *M. sativa* and *B. oleracea* undergoing soaking in (a) APW, (b) NW, and (c) PAW prior to sowing. (d) Subsequent irrigation of seeds that demonstrate nine combinations of seed soaking + irrigation regimes.

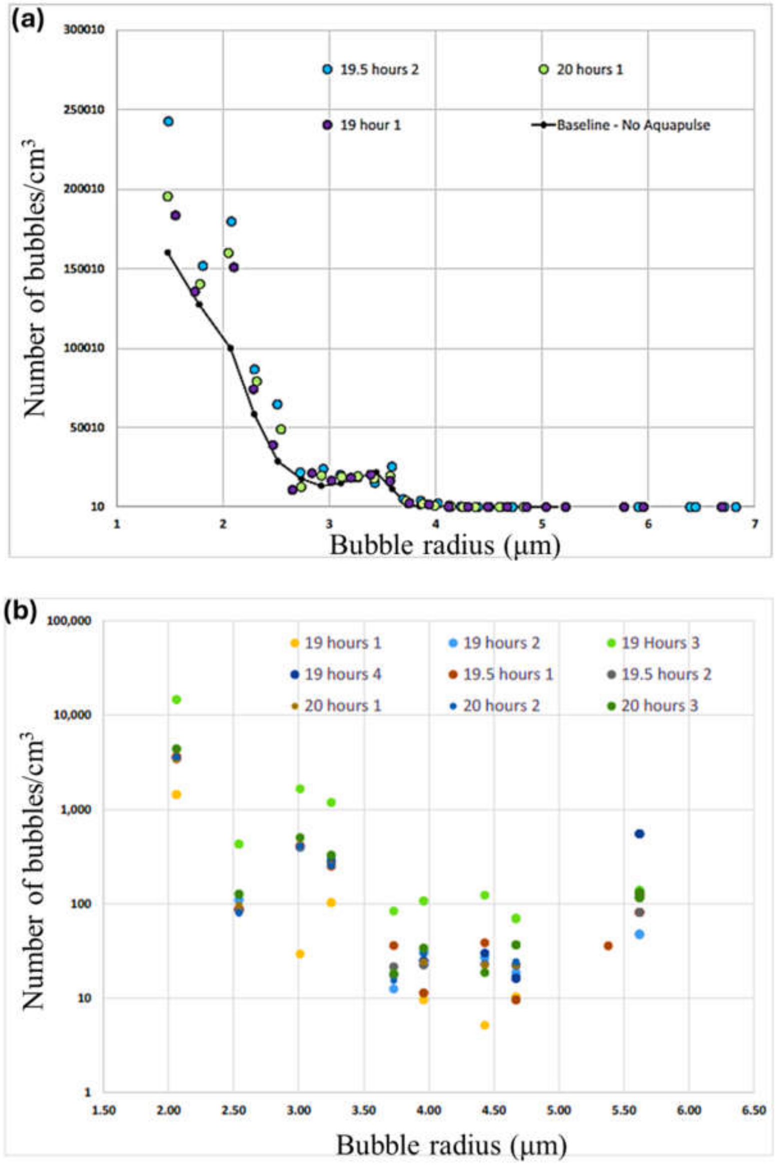

Figure S2. Size distribution of nano-/micro-bubbles generated in APW determined using (a) high-speed video imaging paired with automated image analysis for optical detection and (b) the ABS Acoustic Bubble Spectrometer® for acoustic measurement. Gas bubbles with a *d* of 2–6 μm at concentrations exceeding baseline levels. Baseline data collected in NW prior to feldspar immersion. Measurements taken at 19 h, 19.5 h, and 20 h after introducing the Aquapulse cartridge. Data courtesy of Clear World Water Technology Limited (Letter Report 2M22012, Clear World Water). Study performed by Dynaflow, Inc. (10621-J Iron Bridge Road, Jessup, MD 20794) on behalf of Clear Water Technologies.

Table S1. Concentrations of minerals (in ppm) released from eight different stones present in the Aquapulse® cartridge after 7 days of immersion into 50 mL of deionized water, as determined by ICP-OES using an Agilent 5110 with SPS 4 autosampler. Each pair of rows corresponds to two distinct emission lines for the same element. "u" indicates values below the instrument's limit of quantification.

Element	Wavelength, nm	Sample number							
		1	2	3	4	5	6	7	8
Al	237.312	0.02	0.06	0.00u	0.01	0.01u	0.02	0.06	1.83
	396.152	0.02	0.05	0.00u	0	0	0.02	0.05	1.66
Ca	393.366	5.83	0.45	0.21	0.24	0.29	0.23	1.19	16.67
	422.673	5.12	0.35	0.21	0.23	0.24	0.19	1.51	22.85
Cu	213.598	-0.01u	-0.01u	-0.01u	-0.01u	0.51	-0.01u	-0.01u	-0.01u
	324.754	-0.01u	-0.01u	-0.01u	-0.01u	0.41	-0.01u	-0.01u	-0.01u
Fe	238.204	0	0.03	0	0	0	0.02	0	0.03
	259.940	-0.01u	0.02	-0.01u	0	0	0.02	-0.01u	0.02
K	766.491	0.14	0.48	141.11	78.86	13.30	0.15	0.2	12.64
	769.897	0.21	0.71	88.68	50.66	9.39	-0.47	-0.73u	9.27
Mg	279.553	0.66	0.05	2.78	3.27	0.14	0.02	0.11	0.1
	280.270	0.61	0.05	2.64	3.11	0.13	0.02	0.1	0.09
Na	588.995	0.07	0.25	2.79	3.86	2.24	6.35	0.07	107.39
	589.592	0.01	0.1	1.34	1.85	1.08	3.08	0.01	56.65
P	178.222	-0.01u	0.08	-0.02u	-0.05u	0.17	0.21	0	-0.08u
	213.618	0.04	0.12	0	0	0.17	0.26	0.03	0.01
Si	250.690	0.66	1.27	0.01	0.04	9.69	0.9	0.67	1.35
	251.611	0.67	1.3	0.01	0.02	9.98	0.92	0.68	0.37
Zn	202.548	0	0	0	2.81	0.07	0	0	0.01
	213.857	0	0	0	2.34	0.05	0	0	0.01
